# The visual benefits of correcting longitudinal and transverse chromatic aberration

**DOI:** 10.1167/jov.23.2.3

**Published:** 2023-02-02

**Authors:** Austin Roorda, Steven A. Cholewiak, Swati Bhargava, Nadav H. Ivzan, Francesco LaRocca, Derek Nankivil, Martin S. Banks

**Affiliations:** 1 Herbert Wertheim School of Optometry and Vision Science, University of California at Berkeley, Berkeley, CA, USA; 2 Johnson & Johnson Vision Care, Research & Development, Jacksonville, FL, USA

**Keywords:** longitudinal chromatic aberration, transverse chromatic aberration, chromostereopsis

## Abstract

We describe a system—the Binocular Varichrome and Accommodation Measurement System—that can be used to measure and correct the eye’s longitudinal and transverse chromatic aberration (LCA and TCA) and to perform vision tests with custom corrections. We used the system to investigate how LCA and TCA affect visual performance. Specifically, we studied the effects of LCA and TCA on visual acuity, contrast sensitivity, and chromostereopsis. LCA exhibited inter subject variability but followed expected trends compared with previous reports. TCA at the fovea was variable between individuals but with a tendency for the shift at shorter wavelengths to be more temporalward in the visual field in each eye. We found that TCA was generally greater when LCA was corrected. For visual acuity, we found that a measurable benefit was realized only with both LCA and TCA correction unless the TCA was low. For contrast sensitivity, we found that the best sensitivity to a 10-cycle/degree polychromatic grating was attained when LCA and TCA were corrected. Finally, we found that the primary cause of chromostereopsis is the TCA of the eyes.

## Introduction

### The eye’s chromatic aberration

The index of refraction of the human eye’s ocular media varies inversely with wavelength. Blue rays are refracted more than red. A consequence of this chromatic dispersion is that a black–white edge is not imaged sharply on the retina: It has a color tinge. There are two manifestations of chromatic dispersion in formation of the retinal image: longitudinal chromatic aberration (LCA) and transverse chromatic aberration (TCA). Together, these aberrations adversely affect the image quality of polychromatic stimuli.

LCA is the variation of the eye’s refractive power for different wavelengths. For the human eye, this chromatic difference of focus spans ∼2.5 D from 400–700 nm (Thibos, Ye, Zhang, & Bradley, 1992; Marimont & Wandell, 1994; Atchison & Smith, 2005). The change in refraction (in diopters) as a function of wavelength is well described by an equation from Cauchy (1836) with coefficients specified by Atchison and Smith (2005):

(1) $$\begin{array}{ccc} D(\lambda ) & \;= & 1.6091-\frac{6.7094\times 10^{5}}{\lambda^{2}}+\frac{5.5533\times 10^{10}}{\lambda^{4}}\\ & & \;-\;\frac{5.6000\times 10^{15}}{\lambda^{6}}\; \end{array}$$

where D is the power in diopters, λ is wavelength in nanometers, and 590 nm is in-focus (Atchison & Smith, 2005). LCA follows similar monotonic increases in power from red to blue wavelengths in all humans (Atchison & Smith, 2005), although inspection of individual data reveals nonnegligible variation from one individual to another (Wald & Griffin, 1947; Bedford & Wyszecki, 1957; Ware, 1982; Vinas, Dorronsoro, Cortes, Pascual, & Marcos, 2015). While LCA necessarily degrades image quality for polychromatic light, there is strong evidence that humans use their LCA to aid accommodation (Kruger, Mathews, Aggarwala, & Sanchez, 1993; Cholewiak, Love, & Banks, 2018).

TCA is the variation of image location for different wavelengths. It causes the image of a white point source to be spread across the retina as colored fringes. TCA increases as objects move off-axis (Winter et al., 2016) but is also observed at and near the eye’s best optical axis, including along the line of sight (i.e., at the fovea) (Thibos, Bradley, Still, Zhang, & Howarth, 1990; Simonet & Campbell, 1990b). Because TCA changes with position, it also gives rise to a wavelength-dependent variation of magnification. The magnification difference across the visible spectrum is at most 1% (Thibos et al., 1990). At the fovea, TCA is on the order of minutes of arc, but we will show that it has a discernible effect on visual performance.

Chromostereopsis is an illusion of perceived depth that is generally attributed to TCA. When binocularly viewing small equidistant red and blue objects on a dark background, most people perceive blue as farther than red (Simonet & Campbell, 1990a). TCA causes a horizontal displacement of blue light relative to red. The displacement is usually nasalward on the retina, so it appears temporalward in the visual field. Temporalward displacement causes uncrossed binocular disparity, which is then seen as farther than lights that have less temporalward displacement. The direction of the perceived depth effect is correlated with the direction of TCA displacement in each eye (Simonet & Campbell, 1990a; Ye, Bradley, Thibos, & Zhang, 1991). There is an emerging consensus that chromostereopsis is caused solely by TCA, but it is also possible that perceived blur contributes (Held, Cooper, OBrien, & Banks, 2010; Sprague, Cooper, Reissier, Yellapragada, & Banks, 2016).

### Effects of chromatic aberration on visual performance

It has been known for a long time that visual acuity and contrast sensitivity are better with monochromatic (i.e., narrowband) stimuli than with polychromatic (wideband) stimuli (Luckiesh & Moss, 1933; Campbell & Gubisch, 1967; Yoon & Williams, 2002), a difference that is likely caused by the image degradation that LCA and TCA cause with polychromatic but not monochromatic stimuli. One expects therefore that correcting LCA and TCA would make visual acuity and contrast sensitivity with polychromatic stimuli the same as acuity and sensitivity with monochromatic stimuli. So far, researchers have not been able to demonstrate this. That is, they have found that performance is poorer with polychromatic stimuli with correction of chromatic aberration than with monochromatic stimuli. We believe that this is due to inaccurate correction of LCA and TCA in the previous experiments.

### Research aims

We revisit the effects of chromatic aberration on visual performance by examining whether correcting both LCA and TCA can yield an improvement in visual acuity and contrast sensitivity with polychromatic light. We also revisit whether chromostereopsis is caused solely by TCA or whether there is an additional effect of differential blur. To do the study, we designed and constructed a special apparatus to correct LCA and TCA in both eyes while measuring visual performance. We call it the Binocular Varichrome and Accommodation Measurement System (BVAMS). It has the following features.

(1) Applies variable amounts of correction for LCA, including full and no correction
(2) Applies variable amounts of correction for TCA, including full and no correction
(3) Provides relatively narrowband red, green, and blue primaries at high resolution
(4) Allows independent correction and control of images presented to either eye or both eyes simultaneously
(5) Presents monocular and binocular stimuli across a range of virtual optical distances
(6) Presents binocular stimuli for a range of binocular vergence demands
(7) Measures accommodation while performing near and distance vision tasks (not used in this article)

## Methods

### Hardware

**Figure 1.**
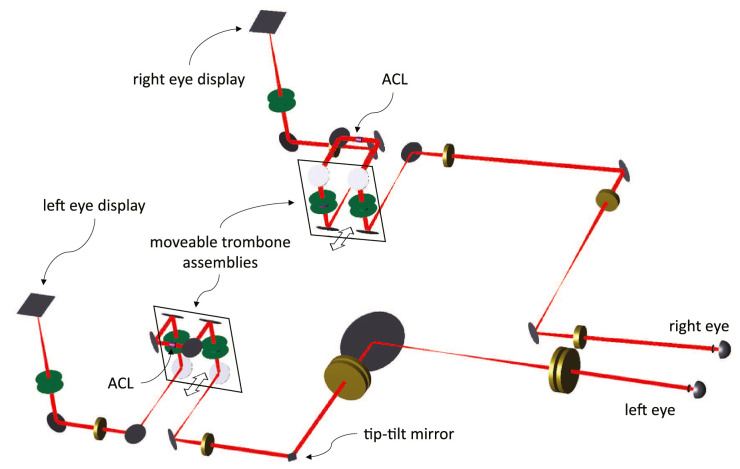
BVAMS optical layout. The Optotune lenses are colored green, the achromatic doublets are colored gold, and the ACLs are colored magenta. The components in the trombone’s moveable assembly consist of four mirrors and two Optotune lenses in the plane of the black squares for both the left- and right-eye subsystems. Everything is drawn to scale, except for the left- and right-eye display sizes and distances from the first Optotunes (both are relatively larger in the actual system).

*General system layout.* The optomechanical design of BVAMS was aided by Zemax (Zemax LLC, Kirkland, WA, USA) and SolidWorks (Dassault Systémes, Waltham, MA, USA) software. The optical layout is shown in Figure 1. Stimuli are displayed on two Active Matrix Organic Light-Emitting Diode (AMOLED) displays (Waveshare 5.5-in. HDMI AMOLED), one for each eye. Optical vergences to the displays are adjusted by the first focus-adjustable liquid lens (model EL-10-30; Optotune, Dietikon, Switzerland) just prior to the entrance pupil. The entrance pupil is a physical 4-mm aperture. The entrance pupil is relayed to the achromatizing lens (ACL) via a first 4f afocal telescope comprising an achromatic doublet and a second Optotune lens. The pupil plane of the ACL is relayed via a second 4f telescope consisting of a third Optotune lens and an achromatic doublet to a stationary mirror in the right eye and a horizontally rotatable mirror in the left eye. Finally, the pupil plane is relayed to the eye pupil by a third afocal telescope consisting of two 1-in. diameter 150-mm-fl achromatic doublets for the right eye and two back-to-back pairs of 2-in. diameter, 300-mm-fl achromatic doublets for the left eye. The diameter of the exit pupil of the BVAMS system is 4 mm. The optical axis of the final components in the left-eye system is turned outward by 5.15${}^{\circ }$ relative to the right eye to enable control of binocular vergence (which is established by adjustments to the left-eye subsystem only). The entire left-eye subsystem is on a translation stage to enable adjustment for interpupillary distance.

**Figure 2.**
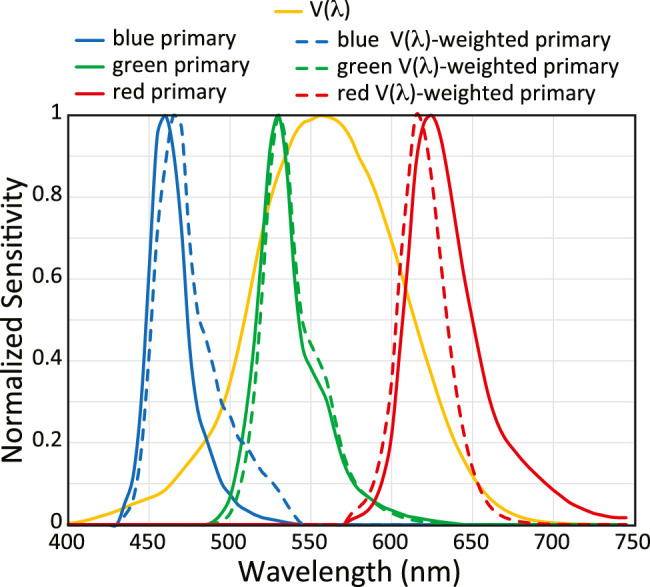
Display primaries and V(λ)-adjusted spectra. The solid curves show the blue, green, and red primaries from the manufacturer. The dashed curves show the same spectra after multiplication with the V(λ) curve (http://www.cvrl.org) and normalization to 1. The median wavelengths of the primaries after multiplication by V(λ) are 468, 533, and 616 nm.

*Display subsystem.* The AMOLED displays are connected via a USB hub to a laptop computer running Microsoft Windows. We chose AMOLED displays for these experiments because of their high contrast ratio, relatively high luminance (∼200 cd/m${}^{2}$ when viewed directly), and the narrowness and separation of the primaries. The spectra of the three primaries are plotted in Figure 2. Solid lines represent the primaries from the manufacturer. Dashed lines show the spectra after multiplication by the photopic luminous efficiency of the human eye (V(λ)) plotted in units of energy (downloaded from www.cvrl.org). The median wavelengths of the three primaries after the multiplication are 468, 533, and 616 nm. An Optotune lens is used to adjust the optical vergence of light from the display prior to the entrance pupil. The range of powers for the lens is 8.3–20 D, so a −13.33-D achromatic doublet lens is placed adjacent to the Optotune lens and between it and the display to make the focal length longer. We did this to maximize the pixel density of the display viewed through BVAMS. Each pixel on the display subtends 0.23 arcmin. The display is at optical infinity with an Optotune setting of ∼14 D (exact unique values were set for the left- and right-eye channels), thereby providing a greater than ±5-D range of optical vergence control. In the system, the Optotune lenses are oriented horizontal to earth to minimize gravity-based aberrations in the lens, as per guidance from the manufacturer (www.optotune.com).

The maximum luminance of the display as viewed through the system is 4.727 cd/m${}^{2}$ (red: 1.348; green: 3.933; blue: 0.3604) in the left eye and 5.265 cd/m${}^{2}$ (red: 1.401; green: 4.109; blue: 0.4064) in the right eye. Gamma correction was done for each primary separately in the two displays. The visible field through the system is circular with a diameter of 3${}^{\circ }$.

Stimuli are rendered with 0.1-subpixel resolution and anti-aliasing by first rendering them in a 10× up-sampled pixel space, then down-sampling them back to display space with linear resampling. Digital TCA offsets were done by adding subpixel offsets to the green and blue primaries relative to the red.

**Figure 3.**
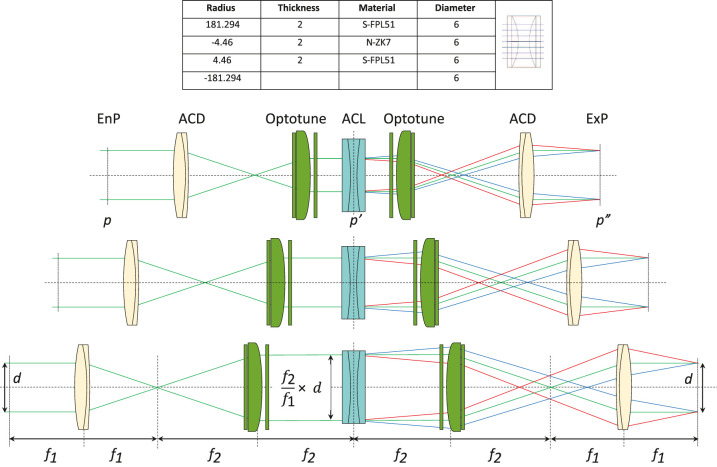
The top panel shows the specifications for the achromatizing lens (ACL). The lower schematics illustrate the principles of the variable LCA correction. Three settings of the optical system are shown. In all cases, the sizes of the entrance (EnP) and the exit (ExP) pupils are kept constant while the beam size at the achromatizing lens ACL is adjusted with a pair of telescopes comprising an achromatic doublet (ACD) and an Optotune lens. From top to bottom, the focal lengths of the Optotune lenses are increased while the telescope distances are adjusted to maintain afocality for green light. The ray diagram shows that by increasing the beam size on the ACL, the magnitude of the LCA correction at ExP is increased.

*Achromatizing lens subsystem.* An ACL was designed by us and custom-built (Optimax, Ontario, NY, USA) for this system. The specifications of the ACL are provided in Figure 3. The ACL is afocal for 573.5-nm light and has positive and negative power for longer and shorter wavelengths, respectively. The magnitude of the LCA was designed to be equal and opposite to that of a typical human eye as provided by Equation 1 (Atchison & Smith, 2005). To adjust the LCA correction in BVAMS, we adjust the magnification of the beam at the ACL relative to the eye using an optical trombone assembly with Optotune lenses. Each arm of the trombone contains an afocal telescope comprising an achromatic doublet and an Optotune lens. The basic principles are illustrated in an unfolded optical layout in the lower part of Figure 3. The doublet and Optotune lenses in each telescope are labeled $f_{1}$ and $f_{2}$ because the powers of the lenses in each pair are kept the same. The change in vergence of a beam between two conjugate points is proportional to the inverse of the magnification between the two conjugate points. Consequently, the LCA at the exit pupil plane in the optical assembly can be manipulated by increasing or decreasing the beam size at the ACL.

Because the vergence of a beam between two pupil conjugates varies by the inverse of the magnification, it follows that the LCA correction at the eye is

(2) $$LCA=\frac{LCA_{ACL}}{M^{2}}=\frac{LCA_{ACL}}{{\left(f_{2}/f_{1}\right)}^{2}}$$

where $LCA_{ACL}$ is the LCA of the ACL in diopters, $f_{1}$ and $f_{2}$ are lens focal lengths and M is magnification (Figure 3). The implementation of the correction using an optical trombone assembly is done by changing $f_{2}$, as illustrated in Figure 1. The quantitative relationship between magnification and LCA correction depends on the LCA of the whole system, including the Optotune lenses; it is described later.

*Binocular vergence subsystem.* The final pupil conjugate in the left-eye system prior to the eye is a rotating mirror (Figure 1). This mirror redirects the beam toward the eye at different angles to provide a binocular vergence. The primary aim is for the binocular vergence to be consistent with the optical vergence set with the display subsystem. For example, a display with a 3-D optical vergence should have a binocular vergence that is consistent with a 33.33-cm viewing distance. To accommodate the largest possible range, the final lenses in the left-eye system are 2 in. in diameter, and the optical axis is turned outward by 5.15${}^{\circ }$. For a 60-mm interpupillary distance, this corresponds to a binocular viewing distance of 666 mm. The 2-in. lens diameters allow for adjustment of binocular vergences ranging from infinity (0 D) to about 25 cm (4 D). Pairs of 300-mm-fl achromatic doublets are used in the 4f telescope rather than a pair of 150-mm-fl achromatic doublets to maintain excellent image quality and a stable exit pupil with changes in binocular vergence. The binocular vergence is adjusted by changing the angle of the exit beam for the left eye only. This keeps the right eye's orientation fixed, which facilitates a future capability of measuring refraction and accommodation simultaneously along the line of sight.

**Figure 4.**
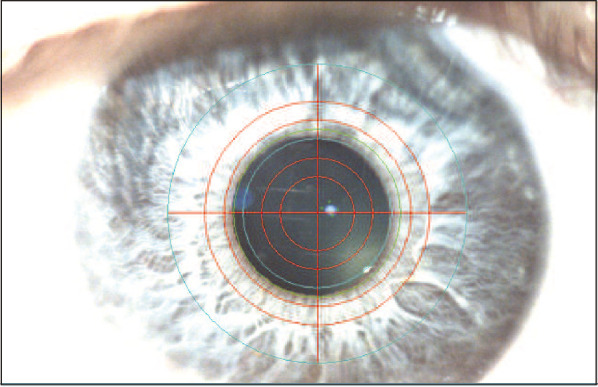
Pupil alignment. Image of the right eye when aligned with BVAMS. Corneal reflex and concentric circular reticle are visible.

*Pupil alignment subsystem.* TCA in a Maxwellian-view system is highly dependent on pupil position (Simonet & Campbell, 1990a; Thibos et al., 1990; Boehm, Privitera, Schmidt, & Roorda, 2019; Domdei, Linden, Reiniger, Holz, & Harmening, 2019), so it is imperative to maintain pupil alignment through the entire procedure. For this purpose, two high-magnification, short depth-of-focus cameras are used to monitor and maintain alignment of both pupils during the task. Each camera had a digital reticle overlay in its display to indicate the exact location of the BVAMS exit pupil. For initial alignment, the eye is illuminated with a desk lamp. The subject uses a bite bar. The right eye is initially aligned using an X–Y–Z translation stage. The axial position is established by moving the eye in the Z direction for best focus of the pupil, and the X–Y position is adjusted to center the pupil on the reticle. The alignment of the left eye is established in a three-step process. The lateral position of that eye is aligned by moving the entire left-eye channel of BVAMS on a translation stage. The vertical position of the left eye is adjusted by changing the roll angle of the bite-bar stage. The axial position of the left eye is adjusted by rotating the bite bar about a vertical axis. The camera images also contain a small reflection of the BVAMS display from the cornea. A digital mark is added to the reticle display at the reflex position in both eyes after alignment is complete. Then the lamp is turned off and the relative alignment of the corneal reflex and digital mark on the display is used to monitor pupil position online during each experiment to monitor and adjust pupil alignment as needed throughout each experiment. Figure 4 shows an image of the pupil with the desk lamps on, the reticles displayed, and the corneal reflex identified.

### System performance

**Figure 5.**
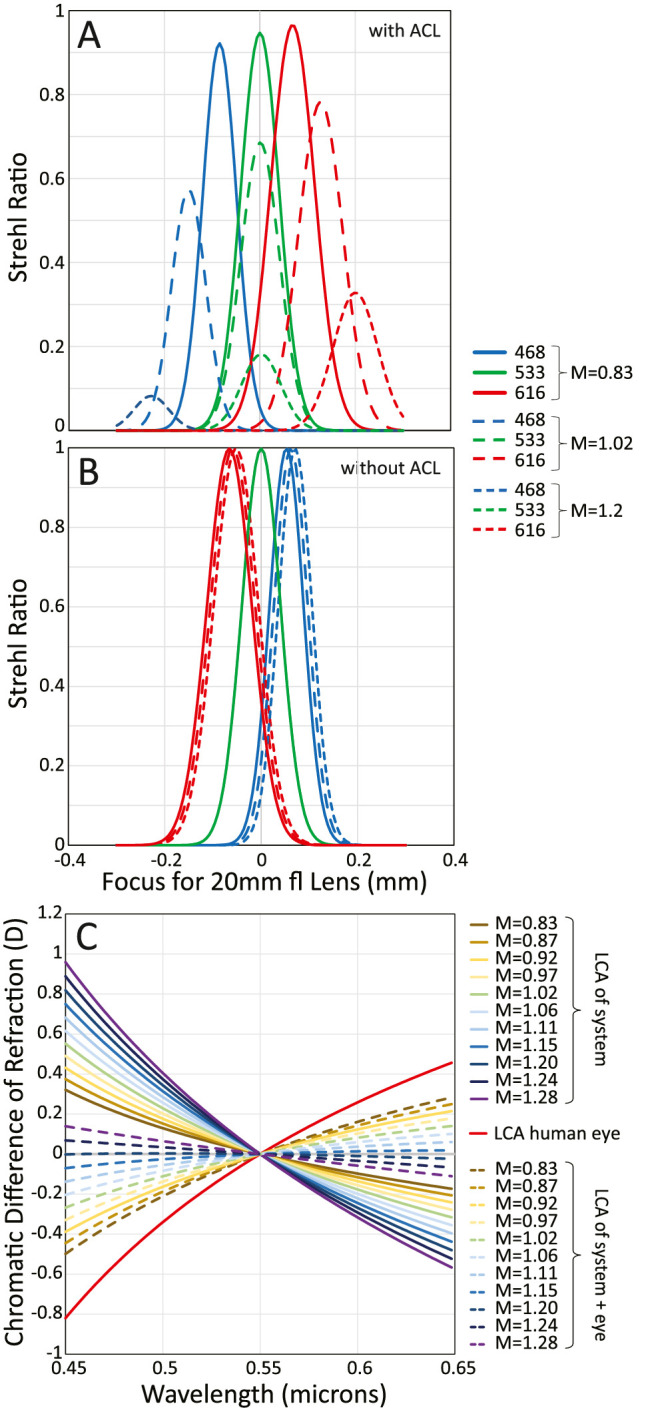
Optical performance of BVAMS. (A, B) Through-focus Strehl ratios for the three primaries at three magnification settings of the trombone. The x-axis indicates shifts from best focus relative to the focus for 533 nm, where positive values indicate that the focus is closer to the lens (i.e., higher power). Panel A shows the Strehl ratios with the ACL in the system, and panel B shows them without the ACL. (C) LCA of BVAMS for various magnifications. The red solid line represents the average LCA of the human eye as in Equation 1.

*System optical quality* . Figure 5 shows optical performance, measured by the Strehl ratio, computed using Zemax at the three primary wavelengths and three magnifications spanning the full range of motion of the trombone. Figure 5A shows performance with the ACL in the system, and Figure 5B shows it without the ACL in the system. The figures also show how the LCA changes as a function of magnification. Note that without the ACL, BVAMS has some positive LCA due to the Optotune lenses. All measures of the eye’s LCA in this report are corrected for this system LCA. As can be seen from Figures 5A and B, optical performance depends on magnification. Performance is nearly diffraction limited at M = 0.83 and a bit poorer at M = 1.20. The decrease in system performance arises primarily from a slight increase in negative spherical aberration in the ACL.

*System LCA.* The Zemax model was used to compute the LCA of BVAMS as a function of magnification. Figure 5C shows the chromatic difference of refraction as a function of wavelength for a range of magnifications. It also shows Equation 1, which represents the LCA of a typical human eye. Finally, the figure shows the difference between the equation and the BVAMS LCA for each magnification. Full correction for a typical eye is obtained with a magnification setting slightly less than 1.2.

**Figure 6.**
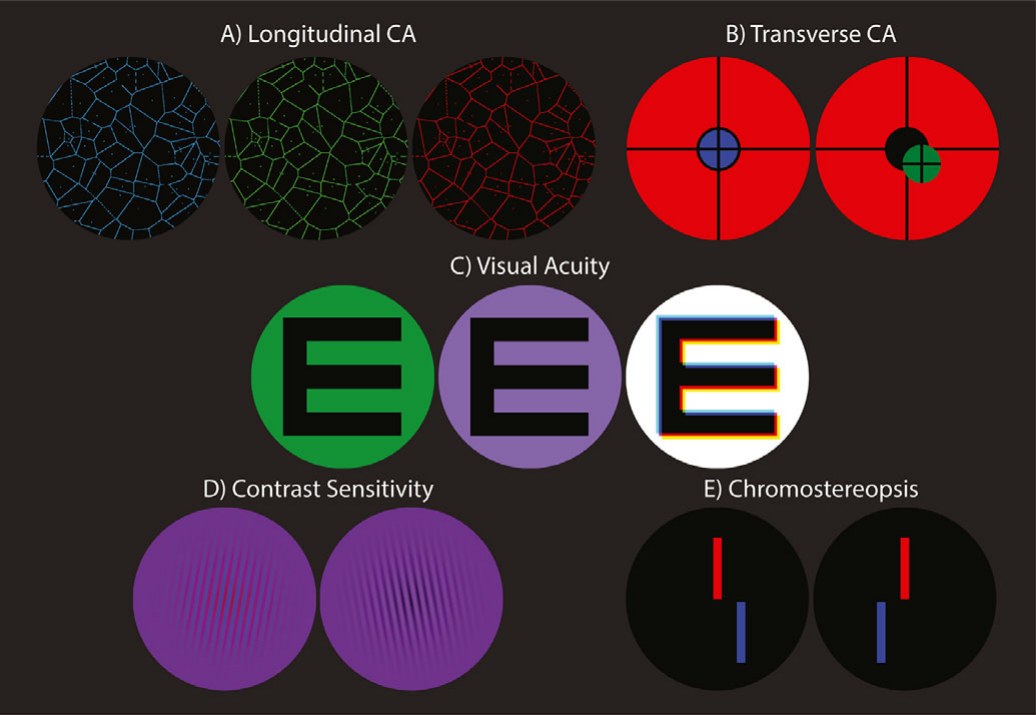
Stimuli for the experiments. (A) Voronoi patterns for LCA measurement. (B) Duochrome disks for TCA measurement. (C) Optotypes for acuity. Left: monochromatic green background. Center: purple background composed of equiluminant blue, green, and red. Right: white background. The optotype in the right panel shows digital TCA offsets. (D) Left- and right-tilted Gabor patches. The left image has red and blue red gratings that are a half wave out of phase. The right image has red and blue gratings that are in phase. (E) Dichoptic red and blue bar pattern with binocular disparity specifying that blue is farther than red when cross-fused.

*System TCA.* BVAMS has a small amount of TCA (never greater than 1 arcmin) due to small misalignments of the beam through the system. We measured the system TCA so that we could correct for it. We did this by placing a high-resolution monochromatic camera where the eye would normally be. We then had the camera view the TCA target used in our experiments (Figure 6B). The camera was carefully centered with the exit pupil in the following way: We set the camera aperture to its smallest diameter and translated the camera vertically and horizontally to center it with the beam. The axis of the camera was set by ensuring that the TCA target was centered on the camera pixel array. System TCA was measured for a range of trombone positions with and without the ACL in place. A lookup table was generated so that the system TCA correction could be applied to the display for any setting of BVAMS and be used to correct all measured TCA.

### Experiments

We conducted six experiments. We list them briefly here and provide the methods and results for each experiment in detail in subsequent sections.

(1) Measured LCA in both eyes.
(2) Measured TCA in both eyes.
(3) Measured letter acuity in one eye under three conditions: dark letters on green, white, or purple backgrounds. We did this with and without LCA correction and with and without TCA correction.
(4) Measured contrast sensitivity in one eye for a 10-cycle per degree (cpd) sinewave stimulus composed of red–black and blue–black gratings added in different phases. We did this with and without LCA correction.
(5) Measured chromostereopsis with and without LCA correction.

### General methods and participants

Participants’ head positions were stabilized with a bite bar and head rest. The tested eye (or eyes) was (were) precisely aligned with BVAMS before any testing began.

Three adults, ages 39, 54, and 73, participated in all the experiments. An additional four, ages 26, 33, 55, and 59, participated in all but the visual-acuity and contrast-sensitivity experiments. The protocol was reviewed and approved by the University of California, Berkeley institutional review board and adhered to the tenets of the Declaration of Helsinki regarding ethical treatment of human subjects for research. Participants provided informed consent before participating. All had normal visual acuity and stereoacuity when wearing their usual optical correction. The older subjects (ages 54, 73, 55, and 59) were presbyopic and therefore could not accommodate to stimuli presented at different optical distances. The three youngest subjects (ages 39, 26, and 33) had their accommodation eliminated via cycloplegia (one drop of 1% tropicamide and one drop of 2.5% phenylephrine).

### Statistics

We used PSIGNIFIT 3.0 toolbox (Fründ, Haenel, & Wichmann, 2011) in MATLAB (The MathWorks, Natick, MA, USA) to fit the psychometric data we collected. For visual acuity, we grouped all the trials to find the 78.1% threshold (280 trials per condition, 4-alternative-forced-choice [4AFC] task). For both the contrast sensitivity and chromostereopsis tasks, we grouped all the trials to find the thresholds for the 2-alternative-forced-choice (2AFC) tasks. We also report 90% confidence intervals.

To explore the significance of the trends observed in the visual-acuity data, we first performed a series of within-subjects analysis of variance (ANOVA) tests. For all tests, the dependent variable was logarithm of the minimum angle of resolution (logMAR) acuity, chosen over MAR because the cumulative histogram of logMAR acuities was normally distributed while that for the MAR acuities was not. The factors were combinations of background color and LCA and TCA correction. ANOVA tests were followed up with two-tailed *t* tests to ask specific questions of the data.

Lastly, to determine how accurately TCA predicted the chromostereopsis data, we used a repeated-measures correlation method (Bakdash & Marusich, 2017).

## Specific methods and results

### Longitudinal chromatic aberration

We measured each participant’s LCA and used those measurements to correct LCA in subsequent experiments.

*Methods.* The stimulus for measuring LCA is shown in Figure 6A. The fine Voronoi pattern was displayed with the red, green, or blue primary on a black background. Participants adjusted the optical distance to the stimulus until the Voronoi pattern was sharpest. They did this by making button presses that caused the power of the display Optotune lens to change. Each button press produced a 0.1-D change. Because monochromatic aberrations were present (including astigmatism), it was not always possible for a subject to make all features of the pattern appear equally sharp. In these instances, subjects were instructed to adopt a consistent criterion for sharpness (e.g., make the vertical lines in the pattern appear sharp) and stick with that criterion for all wavelengths. The three colors were presented in pseudorandom order until six adjustments had been made for each primary.

**Figure 7.**
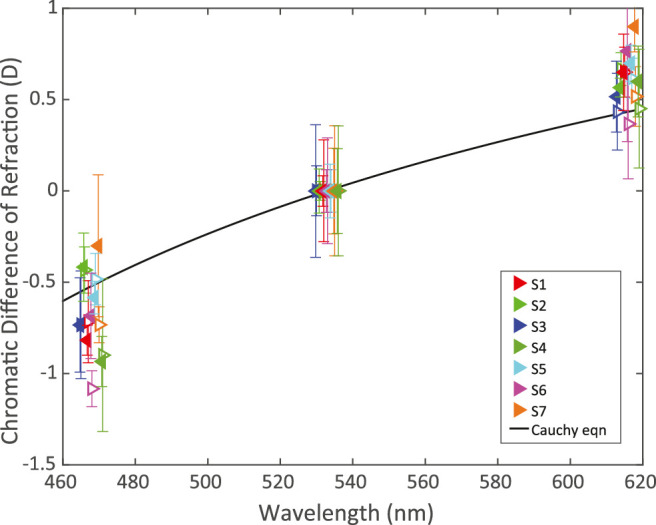
Longitudinal chromatic aberration. Chromatic difference of refraction is plotted for all eyes of all subjects. The values for the green primary (533 nm) have been set to zero. The unfilled triangles pointing to the right are the right-eye data and the filled triangles pointing to the left are the left-eye data. Different colors represent the data from different subjects. Error bars are standard deviations. The data points are slightly shifted horizontally from each other at each primary for clarity. The black line is Equation 1, shifted vertically to be zero at 533 nm. The data for this plot can be found in Table A2.1 in Appendix 2.

*Results.* LCAs for the left and right eyes of all seven subjects are plotted in Figure 7. Equation 1 is plotted for comparison. The refraction for the blue primary (average −0.67 D ± 0.22 D) was ∼1 D greater than that for the red (average +0.60 D ± 0.14 D). The LCA we measured is slightly greater than the model equation predicts, but given the reported variability, it is otherwise consistent with previous reports (Thibos et al., 1992; Marimont & Wandell, 1994; Atchison & Smith, 2005; Jiang, Kuchenbecker, Touch, & Sabesan, 2019).

### Transverse chromatic aberration

We measured each participant’s TCA and used those measurements in subsequent experiments.

*Methods.* The stimulus for measuring TCA is shown in Figure 6B. It was either a blue disk presented within a red annulus or a green disk presented within a red annulus. The diameter of the central disk was 0.5${}^{\circ }$ (130 pixels). The widths of the black lines and gap between the disk and annulus were 2.30 arcmin (10 pixels). Disk positions relative to the annulus were initially random. Participants made button presses to adjust the horizontal and vertical positions of the disk to center it perceptually with the annulus. They could make coarse (1.15 arcmin; 5 pixels) or fine (0.115 arcmin; 0.5 pixel) adjustments as desired. The relative position when the disk appeared centered in the annulus indicates the inverse of the participant's TCA. The blue and green disks were presented in pseudorandom order until six alignments had been done for both. The TCA measurements were done in separate experimental runs with and without LCA correction.

**Figure 8.**
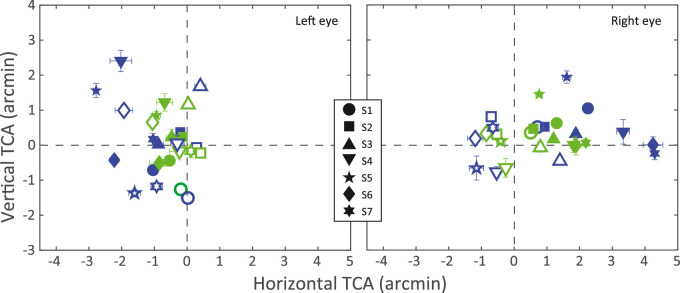
Transverse chromatic aberration. The two panels plot the horizontal and vertical TCA of the blue and green primaries relative to the red. They are the opposite of the offsets each participant required for the disk and annulus to appear aligned and therefore indicate the actual appearance of physically coaligned red, green, and blue objects if the red object appeared at the origin. Positive values on the abscissa and ordinate indicate rightward and upward, respectively. The left and right panels show the data for left and right eyes, respectively. Different symbol shapes represent the data from different participants (as indicated by the legend). Blue symbols represent data for blue relative to red and green symbols data for green relative to red. The filled symbols are the data when LCA was corrected and the unfilled symbols data when LCA was uncorrected. Error bars are standard deviations. The data for this plot can be found in Table A2.2 in Appendix 2.

*Results.* The results are shown in Figure 8, where horizontal and vertical TCA of blue and green are plotted relative to red. The data are also in Table A2.2 in Appendix 2.The values indicate the opposite of the offsets that were required for the disk and annulus to appear aligned. The left and right panels of the figure show the data for the left and right eyes, respectively. Blue symbols are the settings for blue relative to red, and green symbols are those for green relative to red. Filled and unfilled symbols represent the data when LCA was uncorrected and corrected, respectively.

Three effects are evident. First, the blue data deviate more from zero than the green data. This is expected because TCA is greater for short wavelengths relative to long wavelengths than for medium relative to long wavelengths. The median horizontal TCA values for blue were −1.04 and −0.30 arcmin for the left eye (LCA corrected and uncorrected, respectively) and +2.25 and −0.65 arcmin for the right eye; thus, for blue, the average deviation from zero was 1.06 arcmin. The same values for green were −0.54, −0.19, +1.30, and −0.41 arcmin; thus, for green, the average deviation from zero was 0.61 arcmin. In other words, the TCA for blue was nearly twice as large as for green. Our data agree well with those of Rynders, Lidkea, Chisholm, and Thibos (1995), who measured TCA for blue relative to red in the left and right eyes of 85 individuals. Second, the left-eye data exhibit more negative (leftward) horizontal offsets than the right-eye data. The average horizontal TCA for the left and right eyes was −0.52 and +0.62 arcmin, respectively. This too is expected because for most eyes, short wavelengths are seen more temporally than long wavelengths (Simonet & Campbell, 1990a; Rynders et al., 1995). Third, the horizontal TCA values deviate more from zero with LCA corrected than with it uncorrected. The average deviation from zero for corrected LCA was 1.28 arcmin and the average deviation for uncorrected LCA was 0.39 arcmin.

### Visual acuity

As we said earlier, LCA and TCA adversely affect retinal-image quality. Thus, one expects an improvement in visual resolution when these aberrations are minimized or eliminated. Interestingly, no one has demonstrated that correction of these chromatic aberrations for polychromatic stimuli actually yields the expected improvement in visual resolution.

One way to eliminate LCA and TCA is to use a spectrally narrowband stimulus. Luckiesh and Moss (1933) measured grating acuity (i.e., the highest detectable spatial frequency) with monochromatic light (sodium-vapor lamp) and white light (10-watt tungsten-filament lamp). All subjects exhibited better acuity with monochromatic than with white light. The improvement ranged from 24% at low luminance to 8% at high luminance.

Yoon and Williams (2002) investigated the influence of chromatic aberration and higher-order aberrations on visual performance. They examined the effect of higher-order aberrations (e.g., spherical aberration, coma) by using adaptive optics to compare contrast sensitivity with and without correction of those aberrations. They investigated the effect of LCA and TCA by comparing sensitivity with monochromatic light and polychromatic light. Contrast sensitivity improved ∼4-fold when both higher-order and chromatic aberrations were minimized (adaptive optics, monochromatic light) compared to when they were not (conventional optics, polychromatic light). Minimizing LCA and TCA (monochromatic vs. broadband light) while not minimizing higher-order aberrations produced a ∼2-fold improvement. They observed similar effects in a letter-acuity task. The observed effects were consistent with optical theory. They did not compare polychromatic performance with corrected chromatic aberrations to monochromatic performance.

Campbell and Gubisch (1967) also investigated the effect of eliminating LCA and TCA by measuring contrast sensitivity at various spatial frequencies with a monochromatic stimulus and a white stimulus (tungsten lamp). Their data showed consistent improvements in contrast sensitivity with monochromatic relative to white light. One can in principle minimize the effects of chromatic aberration with polychromatic stimuli by using an achromatizing lens to cancel LCA (Hartridge, 1947; Thomson & Wright, 1947). Campbell and Gubisch (1967) used such a lens and measured grating acuity with and without that correction. Surprisingly, they observed no systematic improvement when LCA was corrected. Said another way, eliminating the effect of LCA by use of an achromatizing lens did not improve acuity while eliminating LCA by use of monochromatic light did. The authors attributed the lack of improvement with the achromatizing lens to the possibility that the lens did not eliminate TCA, which of course is eliminated with the use of monochromatic light.

Artal, Manzanera, Piers, and Weeber (2010) used a diffractive optical element to correct LCA and measured visual acuity and contrast sensitivity with and without LCA correction. With LCA correction, the authors did not find an improvement in acuity, but they did find an ∼1.3-fold improvement in contrast sensitivity. Similar to Campbell and Gubisch, TCA was neither measured nor compensated in the Artal et al. study.

Thus, the literature presents conflicting results regarding the impact of LCA correction of polychromatic stimuli, with some demonstrating no improvement in visual performance and others demonstrating a small improvement. Given these conflicting results and limitations, it is desirable to evaluate the visual impact of correcting LCA, TCA, and both. If substantial and consistent improvement can be achieved, it opens the possibility of improving everyday vision with custom-correcting contact lenses or intraocular lens implants.

The primary goal in this project was to determine how correction of chromatic aberration affects visual performance. To this end, we measured visual acuity with corrected and uncorrected chromatic aberrations.

*Methods:* The stimuli for measuring visual acuity are depicted in Figure 6C. They were dark letters E presented on uniform bright backgrounds. The letters were 20% lower in luminance than the backgrounds and were generated using MATLAB and Psychtoolbox-3 (http://psychtoolbox.org/). We used a 4AFC Tumbling-E task with a 2-down/1-up adaptive staircase to adjust the size of the letter trial by trial. Each letter was presented for 500 ms. Participants indicated with key presses whether it was pointing rightward, downward, leftward, or upward. Auditory feedback was provided after each trial indicating whether the response was correct or not. The next letter was displayed once the response from the previous trial had been recorded. Forty trials were conducted in each staircase run. Eight runs were done for each condition, so 360 trials were conducted per subject for each condition in total. A cumulative Gaussian was fit to the resulting psychometric data (proportion correct for each letter size) from the eight runs using PSIGNIFIT, a set of MATLAB functions for fitting psychometric functions (http://psignifit.sourceforge.net/) (Fründ, Haenel, & Wichmann, 2011). From those fits, we determined the threshold acuity (set at 78.1% correct) and confidence intervals.

Acuity was measured in one eye of each participant in four aberration conditions—uncorrected TCA and LCA, corrected TCA and uncorrected LCA, uncorrected TCA and corrected LCA, and corrected TCA and LCA—and three backgrounds—green (approximating monochromatic light), purple (equiluminant red, green, and blue), and white. Figure 6C provides examples of the letters with and without digital correction for TCA on the different backgrounds: green, purple, and white from left to right. The right panel with a polychromatic white background shows an example digital TCA correction, where the blue and green primaries have been shifted down and to the right relative to the red primary: blue twice as much as green. In the experiment, the TCA offsets were custom-corrected for each participant’s measured TCA. Owing to the digital correction method, no TCA correction was made for the green background condition. The LCA corrections were achieved by the trombone setting in BVAMS as described earlier and done according to each subject’s measured LCA.

**Figure 9.**
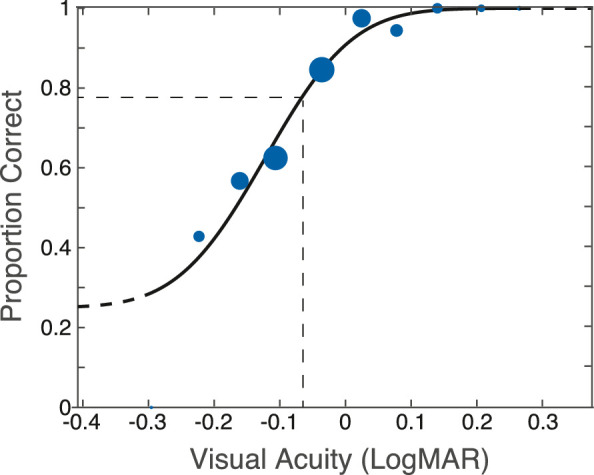
Example psychometric function for the visual acuity experiment. Proportion correct in the 4AFC task is plotted as a function of letter size (in logMAR units). The blue circles represent the data; the diameter of each circle is proportional to the number of trials presented at that letter size. The smooth curve is the best-fitting cumulative Gaussian. Dashed lines indicate 78.1% correct and the corresponding letter size.

**Figure 10.**
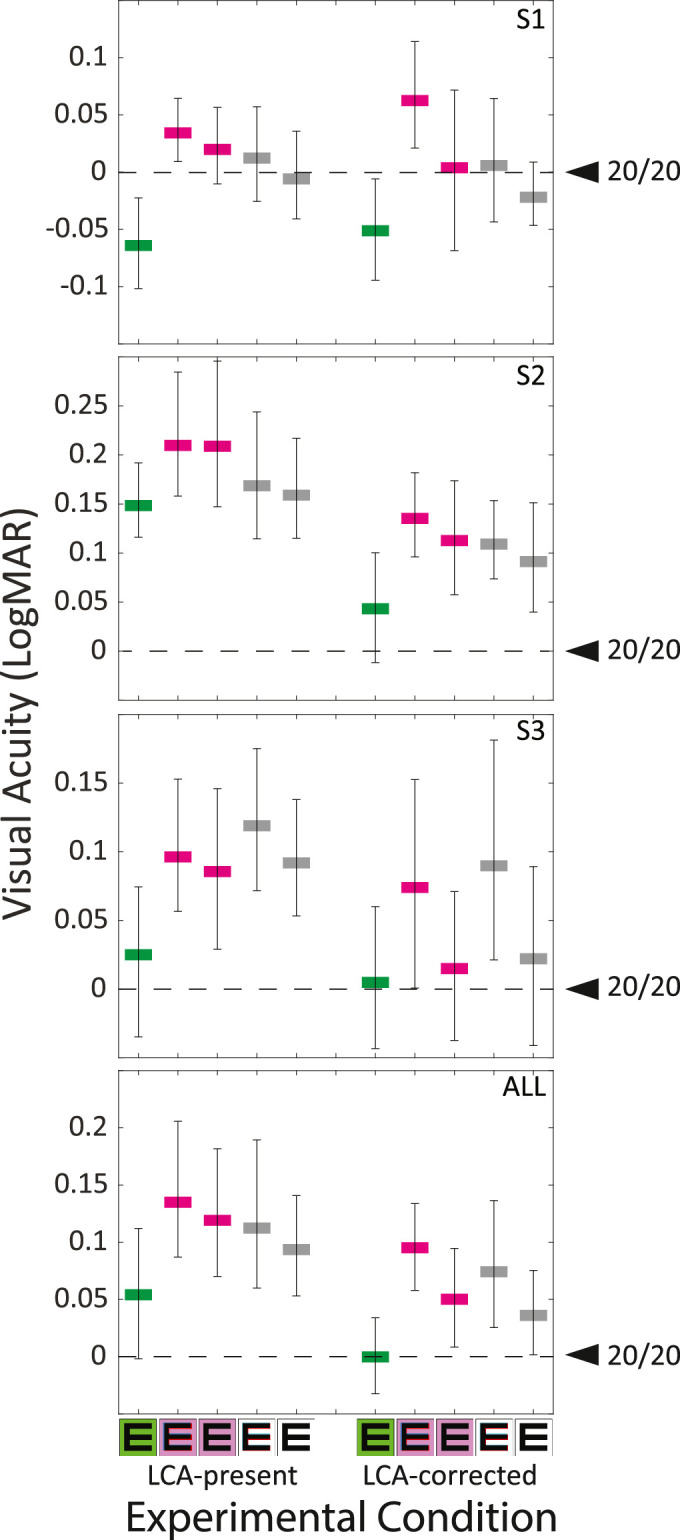
Visual acuity and chromatic aberration. Acuity is plotted as the logMAR for all 10 experimental conditions. Those conditions are indicated by symbols, where the right and left symbols for the purple and white indicate TCA-present and TCA-corrected conditions, respectively. The upper three panels show the results from each of the subjects. The lowest panel shows the average. The average results are plotted in the lowest panel. Error bars are 90% confidence intervals. The data for this plot can be found in Table A2.3 in Appendix 2.

*Results:* Figure 9 provides an example psychometric function and the Gaussian fit to the data. The acuity threshold is indicated by the vertical dashed line. The resulting acuity values are plotted in Figure 10. Each panel shows acuity in logMAR units (the logarithm of the minimum angle of resolution in minutes of arc) for the 10 experimental conditions. The three upper panels show the data individually for each participant. The bottom panel shows the average data. A full 3×2×2 (3 colors, 2 LCA conditions, 2 TCA conditions) ANOVA revealed significant main effects of color (*p* = 0.012) and TCA (*p* = 0.048), as well as a Color×TCA interaction (*p* = 0.028). The full ANOVA, however, did not find LCA alone to be a significant factor. Because Green was a special condition that was not expected to benefit from LCA or TCA correction, we removed it as a color factor and did a 2×2×2 ANOVA. In this test, neither TCA nor LCA on their own were significant factors, but the interaction term of TCA × LCA was (*p* = 0.036). Collectively, these results suggest that, for the small population studied here, both TCA and LCA need to be corrected to yield a visual benefit. This is generally supported by the data plotted in Figure 10. To ask more specific questions and do a deeper dive into individual data, we did a series of *t* tests on each individual and the aggregated group data. The questions and significance of the outcomes of the tests are shown in Table 1. The main outcomes are that visual acuity is better with monochromatic than with polychromatic light and that acuity with polychromatic light is only improved by correcting both LCA and TCA. The only subject who benefited from LCA alone was Subject 2, who had very low TCA before and after LCA correction. In summary, we observed a statistically significant improvement in visual acuity in polychromatic light with correction of chromatic aberrations, but the improvement was small.

**Table 1.** Table of *t* test questions and answers. All *p* values that are bold indicate instances where the answer to the question is YES and the results are significant (p≤0.05). VA = visual acuity.

Question	S1	S2	S3	Group
Q1: Is VA better with monochromatic vs. purple light?	***p* = 0.00005**	***p* = 0.00949**	***p* = 0.0009**	***p* = 0.00392**
Q2: Is VA better with monochromatic vs. white light?	***p* = 0.00614**	*p* = 0.74043	***p* = 0.00104**	***p* = 0.03222**
Q3: Is VA better with monochromatic vs. polychromatic light?	***p* = 0.00004**	*p* = 0.15799	***p* = 0.00015**	***p* = 0.00258**
Q4: Does VA benefit by correcting LCA for monochromatic stimuli?	*p* = 0.40361	***p* = 0.00035**	*p* = 0.36145	*p* = 0.11509
Q5: Does VA benefit by correcting LCA for purple stimuli?	*p* = 0.17474	***p* = 0.0042**	*p* = 0.31651	*p* = 0.19817
Q6: Does VA benefit by correcting LCA for white stimuli?	*p* = 0.7778	*p* = 0.08156	*p* = 0.20702	*p* = 0.12927
Q7: Does VA benefit by correcting LCA for polychromatic stimuli?	*p* = 0.67309	***p* = 0.00196**	*p* = 0.09372	***p* = 0.0466**
Q8: Does VA benefit by correcting TCA for purple stimuli?	*p* = 0.20365	*p* = 0.61315	*p* = 0.3941	*p* = 0.47021
Q9: Does VA benefit by correcting TCA for white stimuli?	*p* = 0.66717	*p* = 0.94499	*p* = 0.3528	*p* = 0.63467
Q10: Does VA benefit by correcting TCA for polychromatic stimuli?	*p* = 0.30348	*p* = 0.70185	*p* = 0.19934	*p* = 0.39105
Q11: Does VA benefit by correcting LCA and TCA for purple stimuli?	*p* = 0.20902	***p* = 0.00073**	***p* = 0.00665**	***p* = 0.00233**
Q12: Does VA benefit by correcting LCA and TCA for white stimuli?	*p* = 0.16139	***p* = 0.03754**	***p* = 0.01694**	***p* = 0.0062**
Q13: Does VA benefit by correcting LCA and TCA for polychromatic stimuli?	*p* = 0.07032	***p* = 0.00018**	***p* = 0.00023**	***p* = 0.00004**
Q14: Is there any difference between VA for monochromatic light vs TCA- and LCA-corrected polychromatic light?	***p* = 0.00256**	*p* = 0.8866	*p* = 0.98751	*p* = 0.26531

### Contrast sensitivity

As we said, our primary goal was to determine how correction of chromatic aberration affects visual performance. Thus, we also measured contrast sensitivity with corrected and uncorrected LCA and various amounts of TCA to determine how sensitivity is affected by correction of both aberrations.

*Methods.* Contrast sensitivity was measured for a stimulus composed of a 10-cpd blue–black sinewave grating and a 10-cpd red–black grating added in different phases (Figure 6D). The peaks and troughs of the blue and red gratings were equal in luminance to one another on the display screen seen through the system. The measurements were done separately with corrected and uncorrected LCA (in the latter case, the peaks and troughs of the two gratings were most likely not equal in luminance at the retina). The focus used for all measurements was the optimal focus for the green primary. In the LCA-corrected case, the correction was done according to each subject’s measured LCA, so the blue and red gratings were also both in best focus. For the LCA-present conditions, the blue and red gratings were both out of focus, with the blue defocus being somewhat greater (see Table A2.1). The gratings were windowed by a Gaussian with a standard deviation of 23 arcmin. The blue grating was horizontally offset from the red by −3, −2, −1, 0, 1, 2, and 3 arcmin, which correspond to phases of -π, $\frac{-2\pi }{3}$, $\frac{-\pi }{3}$, 0, $\frac{+\pi }{3}$, $\frac{+2\pi }{3}$, and +π, respectively. The offset values spanned the full range of the 10-cpd composite. Depending on the offset, the gratings presented variations in hue only (depicted on the left side of Figure 6D) or luminance only (right side). The offsets were presented in pseudorandom order. Measurements of the LCA-corrected and LCA-present conditions were done in separate sessions. Three repeats of each were done for each condition for each subject. TCA between the red and blue primaries (system + subject) was measured prior to each session and was used to compute the actual phase offset of the grating in the retinal image. In a 2AFC task, participants indicated after each stimulus presentation whether the grating was tilted 10${}^{\circ }$ clockwise or counterclockwise from vertical. Auditory feedback was provided. Contrast was modified separately for each offset in 40-trial, 2-down/1-up adaptive staircases. Contrast was increased or decreased by factors of 1.3 according to the staircases. Cumulative Gaussians were fit to the psychometric data using PSIGNIFIT (Fründ, Haenel, & Wichmann, 2011) to determine the contrasts associated with 75% correct and the 90% confidence intervals.

**Figure 11.**
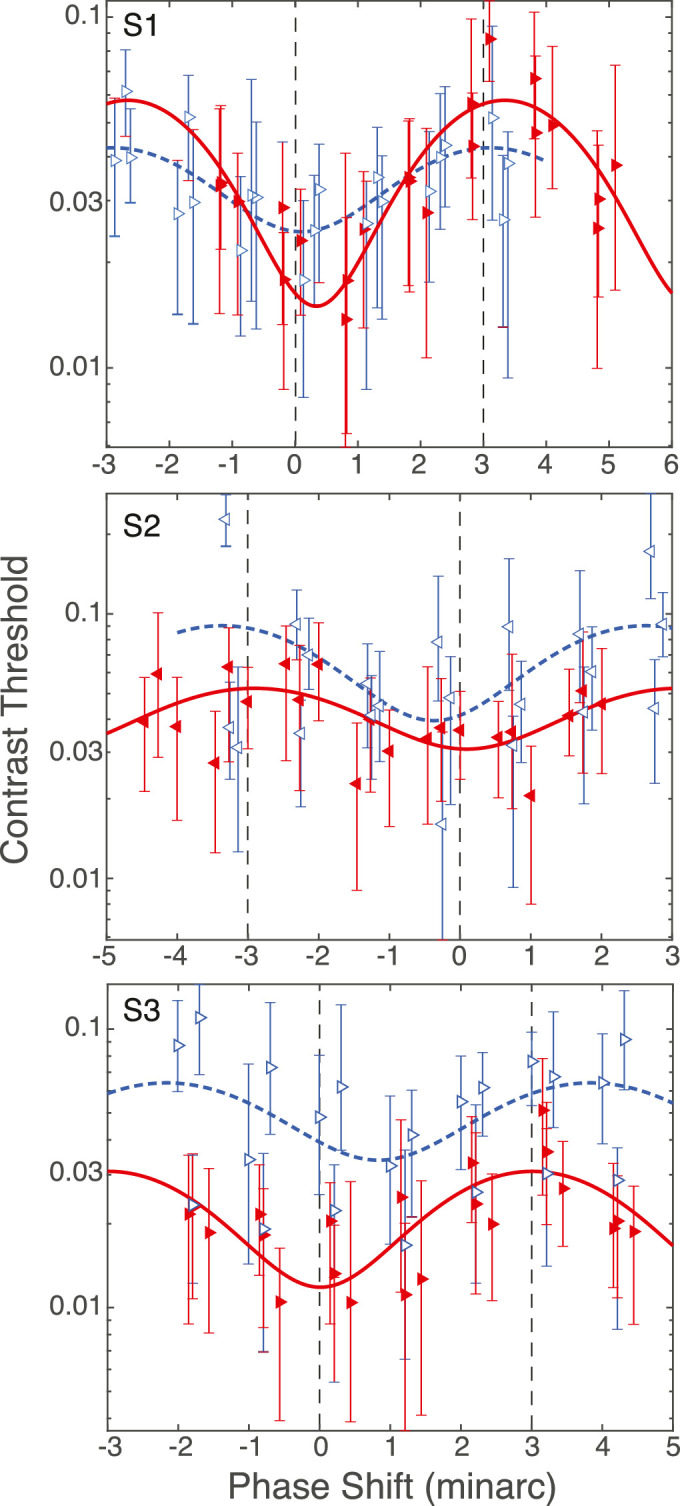
Contrast sensitivity and phase of red and blue gratings. The three panels show the data from the three participants. Each panel plots contrast threshold as a function of the phase of a blue–black grating relative to a red–black grating. A phase shift of zero corresponds to a phase, given the participant’s TCA, that should make the two gratings superimposed on the retina. Shifts of ±3 arcmin correspond to a phase that should make the gratings in counterphase on the retina. 0 and ±3 are indicated by vertical dashed lines. Red filled and blue unfilled symbols represent the data with corrected and uncorrected LCA, respectively. The direction the triangle symbols are pointing indicates the eye that was measured. Error bars are 90% confidence intervals. We fit the data with sinewaves with a period of 2π and free variables for phase, amplitude, and offset. The solid red curve is the fit to the LCA-corrected data, and the dashed blue curve is the fit to the LCA-uncorrected data.

*Results.* Contrast sensitivity to the 10-cpd grating as a function of relative phase is plotted in Figure 11. The abscissa represents the phase shifts (i.e., the phase of the blue grating relative to the red grating). The red filled and blue unfilled symbols represent the data with LCA corrected and LCA present, respectively. There are slight offsets in the TCA-corrected phase shifts between the three repeated trials owing to some variability in the TCA measurements between sessions.

We know from previous work that contrast sensitivity at spatial frequencies higher than 4 cpd is higher for isochromatic, luminance-varying gratings of different colors than for isoluminant, hue-varying gratings with LCA present (Mullen, 1985) or LCA corrected (Zhang, Bradley, & Thibos, 1991). Therefore, we expect thresholds to be lowest for a relative phase shift of zero on the plots, which corrects for the participant’s (and system’s) TCA and creates a grating varying only in luminance. To help find the phase at which contrast threshold was lowest, we fit the data with sinewaves with a period of 2π:

(3) $$\begin{array}{c} c\left(x\right)=a\sin\left[2\pi \left(\frac{x}{6}-p\right)\right]+b\; \end{array}$$

where c(x) is contrast threshold and a, b, and p are free parameters for modulation amplitude, vertical offset, and phase shift, respectively.

There are two main observations.

First, the plots show that the lowest thresholds (maximum sensitivity) are indeed reached when the highest contrast, purest luminance grating is projected onto the retina. This condition occurs when both LCA and TCA are corrected (phase shift = 0 on the red curve of Figure 11). If TCA but not LCA is corrected (phase shift = 0 on the blue curve of Figure 11), the thresholds are not as low, owing to contrast-reducing defocus in the red and blue primaries.

Second, the thresholds vary in sinusoidal fashion with phase shifts between the red and blue gratings, reaching their highest point (lowest sensitivity) when the phase shifts are 180${}^{\circ }$. With LCA correction, this represents a near-perfect isoluminant red–blue chromatic grating. When LCA is not corrected, this represents the closest approximation to an isoluminant grating, although different levels of blue and red defocus leave some residual luminance variation. For all three subjects, the highest thresholds always occurred at or near the isoluminant condition, but whether they occurred for the LCA-corrected or LCA-present conditions was mixed.

### Chromostereopsis

Chromostereopsis is an illusion of perceived depth between objects of differing colors. Specifically, when equidistant short- and long-wavelength objects are viewed binocularly against a dark background, long-wavelength objects are usually perceived as nearer than short-wavelength objects (Einthoven, 1885; Kishto, 1965; Sundet, 1978). Chromostereopsis is usually attributed to a binocular disparity induced by horizontal TCA in opposite directions in the two eyes (Einthoven, 1885; Simonet & Campbell, 1990a; Ye et al., 1991). For example, a temporal offset of blue relative to red in both eyes creates an uncrossed binocular disparity at the retinas specifying that blue is farther than red. Ye et al. (1991) tested this idea quantitatively. They placed 1-mm artificial pupils in front of each eye and displaced them in opposite directions from the eyes’ achromatic axes. They measured the perceived offset of blue relative to red in each eye separately for different pupil positions. They then used those measurements to predict the disparity created when viewing the blue and red objects binocularly. There was excellent agreement between predicted and observed disparity showing that TCA does in fact account for the chromostereopsis effect with pinhole viewing.

But some researchers have reported that the illusory depth percept also occurs with monocular viewing (Donders, 1886; Karkowski & Lloyd, 1951; Over, 1962). They attributed the monocular effect to accommodation. Short wavelengths are, of course, generally focused in front of the retina and long wavelengths behind. Thus, to bring blue into focus, the eye must exert negative accommodation (relax) as it would to focus a truly farther object. And to bring red into focus, the eye must exert positive accommodation as it would with a nearer object. These researchers hypothesized that proprioceptive signals from the ciliary muscle inform distance estimates and therefore create the illusory depth difference.

It is also possible that blue is seen as farther than red because its retinal image is generally blurrier than red. The eye typically accommodates to medium wavelengths (Bobier, Campbell, & Hinch, 1992). Because LCA is greater at short wavelengths relative to medium than for long wavelengths relative to medium (Thibos et al., 1992; Atchison & Smith, 2005), the images of blue objects are usually blurrier than red. In the natural environment, objects that create blurred retinal images are more likely to be farther than objects that create sharp images (Held et al., 2010; Sprague et al., 2016). The visual system uses this statistical regularity to infer that blurriness means farther (Sprague et al., 2016). It is important to note that the experiments of Ye and colleagues (1991) do not enable a test of the accommodation and blur hypotheses because they used pinhole pupils, which means that the retinal images for blue and red were equally sharp. Thus, we used one of the unique capabilities of BVAMS to determine whether chromostereopsis under more natural viewing conditions is wholly attributable to TCA or whether it is also attributable to differential blur. We could not test for an effect of accommodation because our participants could not accommodate during the measurements: the younger ones because they were cyclopleged and the older ones because they are presbyopic.

*Methods.* Chromostereopsis was measured using a binocular stimulus composed of blue and red vertical bars (Figure 6E). Trials were initiated by the participant making a key press. Stimulus duration was 1 s. The blue and red lines were 2.30 × 60.0 arcmin; the gap between them was 2.30 arcmin. Participants indicated after each stimulus presentation whether the upper or lower bar appeared nearer. The binocular disparity of the blue line relative to the red was varied from trial to trial according to the method of constant stimuli. Nine disparities were presented in random order until 10 judgments had been made for each. The psychometric data (proportion of trials in which blue was reported as nearer as a function of disparity) were fit with cumulative Gaussians using PSIGNIFIT (Fründ, Haenel, & Wichmann, 2011). The disparity giving rise to equal perceived depth was defined as the 50% point on the fitted function.

We conducted two stereopsis experiments: one with LCA corrected (custom for each subject) and the other with LCA present. When LCA was present, the blue line was noticeably blurrier than the red line. When LCA was corrected, the blue and red lines were both sharp. If TCA provides a full account of chromostereopsis, the measured TCA will provide an accurate prediction of the disparity that yields equal perceived depth, whether LCA is corrected or not. But if target blurriness also has an effect, the accuracy of the TCA predictions will be more accurate with LCA corrected than with it uncorrected.

The same seven individuals who participated in the LCA and TCA experiments also participated in the chromostereopsis experiment.

**Figure 12.**
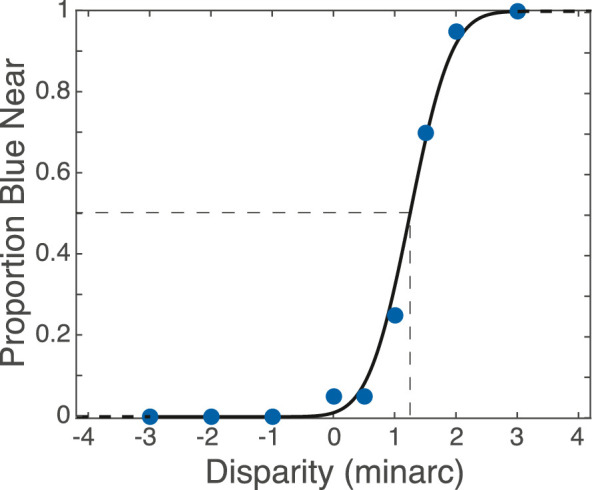
Example psychometric function in the chromostereopsis experiment. The proportion of trials in which the participant reported that the blue line appeared nearer than the red line is plotted as a function of the disparity of the blue line relative to the red. Positive disparity corresponds to crossed (near) disparities for blue relative to red. The blue circles represent the data. The smooth curve is the best-fitting cumulative Gaussian. Dashed lines indicate the 50% point and the corresponding disparity.

*Results.* Figure 12 provides an example of the psychometric data generated by one participant in one condition. The proportion of trials for which the subject reported that the blue line appeared nearer than the red one is plotted as a function of the disparity of the blue line relative to the red. Positive disparity corresponds to increasing crossed (near) disparity of blue relative to red. The dashed lines indicate the value corresponding to blue and red appearing equidistant (i.e., disparity at 50%).

We asked whether each subject’s chromostereopsis effect (i.e., the disparity that makes blue and red appear equidistant) is predicted by their horizontal TCA. The predicted disparity d is

(4) $$d=TCA_{R}-TCA_{L}$$

where $TCA_{R}$ and $TCA_{L}$ are the horizontal components of the TCA measured in the TCA experiment.

**Figure 13.**
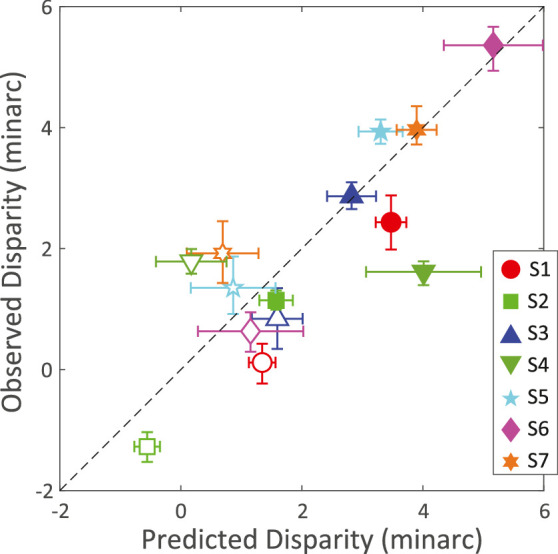
Predicted and observed chromostereopsis. The disparity for which blue and red appeared equidistant is plotted against the disparity predicted from TCA measurements. Different symbol types represent the results from different participants. Filled symbols represent the results when LCA was corrected and unfilled symbols the results when LCA was not corrected. The horizontal error bars represent the magnitude of the left and right standard deviations. The vertical error bars are the 90% confidence intervals from the psychometric data. The data for this plot can be found in A2.4 in Appendix 2. Note that the TCA values differ from those listed for the same subjects in A2.2 because the values reported here include the system TCA.

The results are shown in Figure 13. The horizontal axis is the predicted disparity of blue that should appear equidistant to red if TCA is the only determinant of the chromostereopsis effect (Equation 4). The vertical axis is the disparity that actually made blue and red appear equidistant. The filled and unfilled symbols are the predictions and data when LCA was corrected and not corrected, respectively. If the predictions from TCA were perfect, the data would lie along the diagonal dashed line. As you can see, the data conform quite well to this prediction: The correlation between observed and predicted is highly significant (*r* = 0.812, *p* = 0.00042).

If there were an additional effect of blur (specifically, the blurrier line appearing farther than the sharper line), the data should differ in the LCA-corrected and LCA-present conditions. In the uncorrected condition, the blue line appeared blurrier than the red, so by the blur hypothesis (Held et al., 2010; Sprague et al., 2016), it should have appeared additionally farther in the uncorrected than in the corrected condition. In other words, the data should deviate more rightward from the prediction line (more crossed disparity) in the uncorrected condition than in the corrected one. We computed the horizontal differences between predicted and observed for the two conditions and found that the average values were −0.017 and +0.418 arcmin for the uncorrected and corrected conditions, a very small difference that does not approach statistical significance and is in the incorrect direction anyway.

We conclude that TCA is an accurate predictor of the chromostereopsis effect in natural viewing conditions (uncorrected LCA with natural pupils) and in unnatural conditions (corrected LCA with natural pupils), thus adding to the consensus that chromostereopsis is caused entirely by TCA.

## Discussion

BVAMS is a powerful optical device for measuring and correcting chromatic aberration of the human eye and then assessing visual performance with and without such correction. Here we review our findings, compare them to previous work, and consider implications.

*LCA follows expected trends.* Our measurements of LCA are close to those specified by the Atchison and Smith (2005) model, but we observed consistent and repeatable differences across our subjects. In previous reports, LCA was averaged across subjects (Bedford & Wyszecki, 1957; Vinas et al., 2015; Wald & Griffin, 1947; Ware, 1982), which obscured variation between individuals. We conclude that LCA varies nontrivially from one person to the next, which means that attempts to correct LCA should be preceded by measurements in individuals.

*TCA depends on conditions.* To our knowledge, we made the first measurements of TCA with and without LCA correction. The measurements were very repeatable but differed according to whether LCA was corrected or not: Measured TCA was greater when LCA was corrected than when it was not corrected. Why might this be the case? TCA changes with pupil shifts (Boehm et al., 2019; Zhang et al., 1991; Simonet & Campbell, 1990a), but pupil position was fixed in our experiment, so one might expect TCA to be the same with and without LCA correction. However, perceived chromatic offsets are known to depend on aberrations and blur: TCA estimates are more variable when monochromatic aberrations are present (Boehm et al., 2019; Marcos, Burns, Prieto, Navarro, & Baraibar, 2001) and are generally found to be greater after monochromatic aberrations are corrected (Aissati, Vinas, Benedi-Garcia, Dorronsoro, & Marcos, 2020). Thus, it is not surprising that measured TCA changed between conditions in our experiment.

*Visual acuity benefits of LCA and TCA correction.* Table A2.3 summarizes the statistical analysis of the results for each individual and for the group average. The main findings are the following. (1) Acuity is better with monochromatic light than with polychromatic light whenever LCA and TCA are present. (2) The benefits of LCA correction alone or TCA correction alone do not consistently yield benefits in acuity: A full correction of LCA and TCA is required. That said, VA was improved by correction of LCA alone for polychromatic stimuli for the group as a whole (Q7 in Table 1), but there was no improvement by correction of TCA alone (Q8 in Table 1). This indicates that correction of LCA improves vision more than correction of TCA. That conclusion is further supported by the modeling in Appendix 1. (3) When LCA and TCA are both corrected, visual acuity with polychromatic light is not worse than acuity with monochromatic light (Q14 in Table 1).

There is a potentially important optical factor that may have confounded our ability to measure differences between LCA-present and LCA-corrected conditions in BVAMS. The ACL has inherent monochromatic aberrations. Those aberrations increase with the beam size and, consequently, with the magnitude of LCA correction. The ACL-induced aberrations are coherently coupled with the eye’s aberrations, so the net result may be an increase or a decrease in the aberration experienced by the observer. We did not control for the ACL-induced aberrations and we did not measure the monochromatic aberrations of our subjects. Despite their unquantified effects, they did not preclude our ability to test the effects of LCA and TCA on individual performance, nor did they prevent us from achieving excellent visual performance after correcting LCA and TCA with polychromatic light: performance that was statistically indistinguishable from monochromatic performance.

*Contrast sensitivity is best when TCA is corrected.* We presented red–black and blue–black gratings added in different phases to determine how TCA and LCA correction affected contrast threshold. There were two results. First, highest contrast sensitivity occurs when the phase offset corrects for the eye’s TCA. Second, the effect of TCA correction is greater when LCA is corrected than when it is not corrected.

*Chromostereopsis is minimized when TCA is corrected.* We performed the first test of whether TCA alone is sufficient to explain chromostereopsis, or whether differential blur also contributes. We found a strong correspondence between the disparity required to neutralize chromostereopsis and the disparity predicted from TCA alone. We found no evidence for an effect of blur. Thus, TCA is sufficient to account for the illusory depth between short- and long-wavelength lights, as has been suggested before (Einthoven, 1885; Simonet & Campbell, 1990a; Ye et al., 1991).

*Expected performance improvement with correction of chromatic aberration.* Chromatic aberration varies in the population (Wald & Griffin, 1947; Bedford & Wyszecki, 1957; Charman & Jennings, 1976; Ware, 1982; Howarth & Bradley, 1986; Thibos et al., 1992; Vinas et al., 2015; Jiang et al., 2019; Suchkov, Fernandez, & Artal, 2019; Simonet & Campbell, 1990b; Rynders et al., 1995). Many people have negligible TCA at the fovea, but some have nonnegligible amounts (Rynders et al., 1995). Most of those with nonnegligible amounts have temporal shifts of short-wavelength light compared to long-wavelength light, but some have nasal shifts (Rynders et al., 1995). Everyone has substantial LCA with greater refractive power for short wavelengths than for long, but magnitude varies across individuals (Wald & Griffin, 1947; Bedford & Wyszecki, 1957; Charman & Jennings, 1976; Ware, 1982; Howarth & Bradley, 1986; Thibos et al., 1992; Vinas et al., 2015; Jiang et al., 2019; Suchkov et al., 2019). We investigated the expected effect of correcting chromatic aberration on visual performance by calculating the area of the photopic efficacy–weighted modulation transfer function (MTF). The influence of large and small amounts of LCA and TCA together and in isolation was evaluated. We found that the impact of physiologically plausible amounts of LCA was greater than that of plausible amounts of TCA. The analysis is described in Appendix 1.

*Adaptation for chromatic aberration.* As noted earlier, previous experiments in which LCA was corrected observed little to no improvement in visual performance (Luckiesh & Moss, 1933; Campbell & Gubisch, 1967; Yoon & Williams, 2002; Artal et al., 2010). Some have argued that the lack of improvement was due to inaccurate correction of LCA and/or TCA (Howarth & Bradley, 1986). Others have argued that no clear improvement is expected once one considers the likely effects of such correction on retinal-image quality (Bradley, Zhang, & Thibos, 1991; Thibos, 1987). Yet others have suggested that the absence of substantial improvement reflects a neural compensation for habitual chromatic aberration (Suchkov et al., 2019; Fernandez, Suchkov, & Artal, 2020).

Is there experimental evidence indicating that neural compensation for TCA and/or LCA exists? There is strong evidence against such a mechanism for habitual TCA. Objective and subjective measurements of TCA were made in the same subjects (Harmening, Tiruveedhula, Roorda, & Sincich, 2012). Objective measurements were done by recording relative displacements in the retinal image of three different wavelengths. Subjective measurements were done by having the subjects adjust the positions of stimuli with those same wavelengths until they appeared aligned. The measured TCA differed substantially across subjects, whether the measurements were done objectively or subjectively. But the objective and subjective measurements were essentially identical to one another within subjects. If neural compensation for a subject’s habitual TCA had occurred, the subjective data would have exhibited consistently smaller displacements between wavelengths than the objective data. The fact that this did not occur is evidence that no neural compensation for TCA had occurred.

What about compensation for LCA? The results from our visual acuity experiments suggest that no such compensation occurs, or at least, if compensation occurs, it is not sufficient to completely override the adverse effects of LCA. Specifically, we observed a small but consistent improvement with polychromatic stimuli; indeed, the polychromatic acuities with LCA and TCA correction nearly equaled the monochromatic acuity and were statistically no different as a group (Q14 in Table 1). If neural compensation for habitual LCA had occurred, one would not expect that correcting LCA would yield better acuities. However, our optical simulations (Appendix 1) indicate that chromatic aberrations reduce the area under the MTF substantially, even for a subject with significant amounts of monochromatic aberrations. Given prior work (Atchison, Smith, & Efron, 1979) on the role of uncorrected refractive error on visual acuity, as well as the relatively large reduction in the area of the MTF caused by chromatic aberrations, one may have expected chromatic aberrations to have had a more deleterious effect. Thus, more work is needed to explore this topic and better characterize the expected influence of chromatic aberration on visual acuity and to determine whether neural compensation for chromatic aberration exists.

We also conducted some pilot testing to look for manifestations of neural compensation for LCA and TCA. We presented two stimuli simultaneously and had subjects report which of the two had greater perceived fringing. One of the stimuli had no correction for LCA or TCA (that is, the subject saw the effects of their native LCA and TCA), and the other had some combination of full correction of the two aberrations. Subjects consistently reported that the stimuli with native aberrations had more apparent fringing, which is the opposite of what one would expect if neural compensation for habitual chromatic aberrations had occurred.

We conclude that previous data and our current data do not provide evidence for neural mechanisms that work to minimize the effects of habitual LCA and TCA.

*Benefits of correcting chromatic aberration.* We found a consistent but small improvement in visual acuity and contrast sensitivity when LCA and TCA were both corrected. The improvement was greater with LCA correction than with TCA correction. One naturally asks whether there is a potential eye-care benefit in correcting chromatic aberration with a contact lens or an intraocular lens implant. Consider correcting LCA: Although there is a potential benefit for visual performance, there is also a possible cost. LCA provides a crucial signal for guiding accommodation (Kruger et al., 1993; Cholewiak, Love, Srinivasan, Ng, & Banks, 2017, 2018). For example, neutralizing or reversing LCA has a clear detrimental effect on accommodative responses (Kruger et al., 1993), and erroneous responses would harm visual acuity and contrast sensitivity. We note, however, that the evidence for an important role of LCA in guiding accommodation was obtained with monocular stimulation and an eye-care solution would surely be binocular, in which case binocular vergence could guide accommodation. More research is required to determine if the vergence signal would be sufficient to make up for the loss of the LCA signal. In any event, the best population for correction of chromatic aberration might be middle-aged and older people who have little or no accommodation. If the correction were in the form of a contact lens, movement of the lens would cause variation and probably increases in TCA (Zhang et al., 1991), which would in turn be detrimental to visual acuity and contrast sensitivity. If the correction were in the form of an intraocular lens implant, one would want to ensure that TCA is minimal along the line of sight; otherwise, the correction might not yield any performance benefit.

## Conclusion

Longitudinal and transverse chromatic aberrations have deleterious effects on vision. Using BVAMS, we found that correcting both LCA and TCA in the human eye yields small but measurable improvements in visual acuity and contrast sensitivity, and it eliminates illusory depth between short and long wavelengths along the line of sight. Correcting LCA alone yields visual benefit depending on the magnitude of a person’s TCA.

## Figures and Tables

**Figure A1.1.**
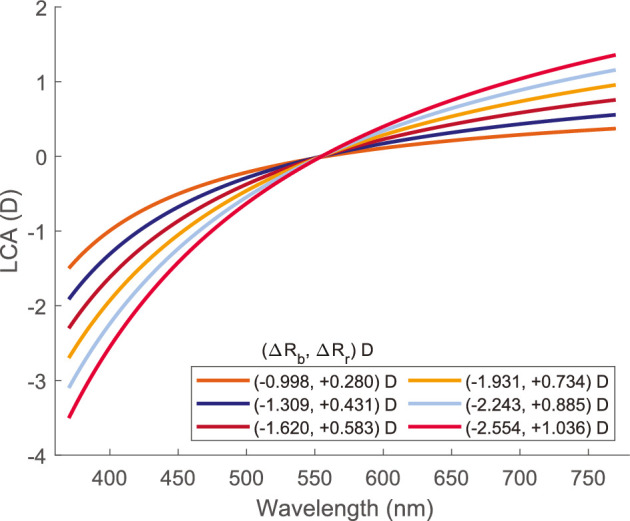
Model demonstrating a range of plausible LCA in the human eye. The legend indicates the ordered pair (Δ$R_{b}$, Δ$R_{r}$), with the axial refractive error in the blue (λ = 400 nm) and red (λ = 700 nm) region of the spectrum, used to solve the system of Equation A1.5 to obtain parameters a, b, and c, describing the variation of refractive index with wavelength. Note that Δ$R_{g}$ = 0 such that the axial refractive error for green light (λ = 555 nm) is zero for all curves.

**Figure A1.2.**
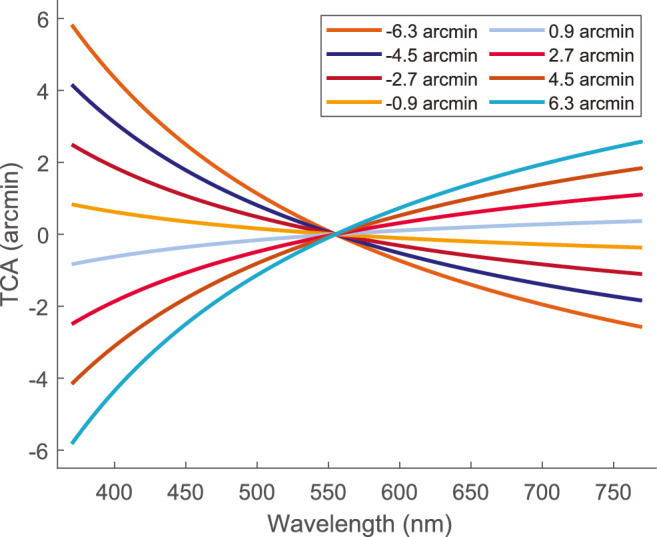
Model demonstrating a range of plausible TCA found in the human eye. The legend indicates the total TCA, δ = γ${}_{r}$ – γ${}_{b}$ for the red (λ = 7,000 nm) and blue (λ = 400 nm) region of the spectrum, used to solve Equation A1.12 for ε. Note that γ(λ = 555 nm) = 0, such that TCA for green light is zero for all curves.

**Figure A1.3.**
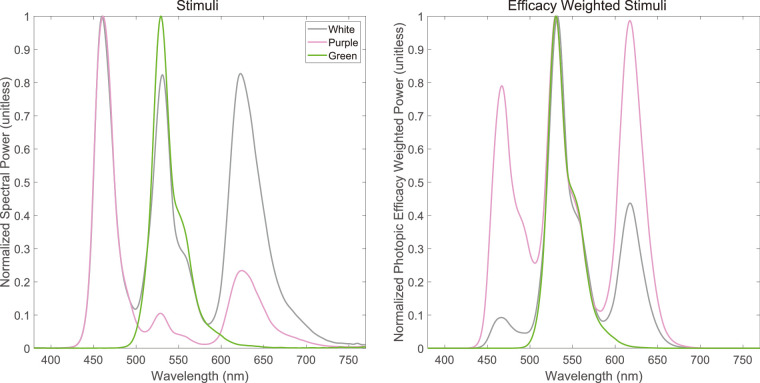
Stimuli spectral characteristics. Normalized spectral power (left), photopic efficacy–weighted spectral power (right).

**Figure A1.4.**
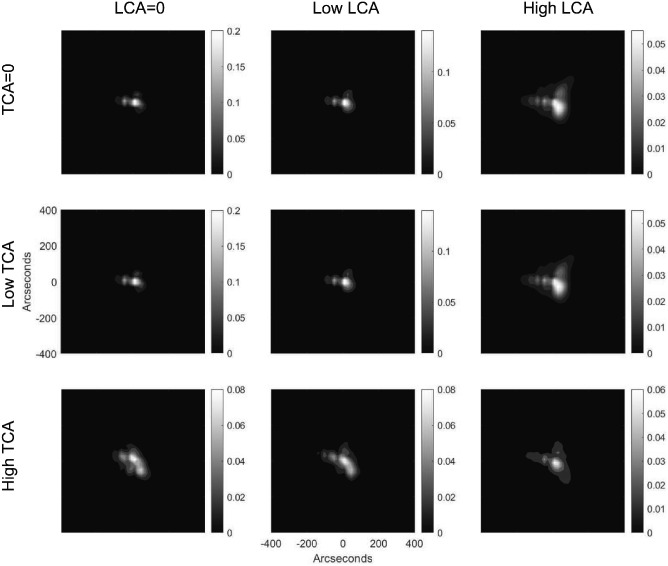
Polychromatic PSF for the white stimulus for one typical subject with increasing amounts of LCA and TCA. Low LCA = −0.998, +0.280 D, and high LCA = −2.554, +1.036 D at λ = 400, 700 nm. Low TCA = 0.4, 0.2 arcmin, and high TCA = 6.3, 3.1 arcmin in the sagittal and transverse meridians from λ = 400-700 nm.

**Figure A1.5.**
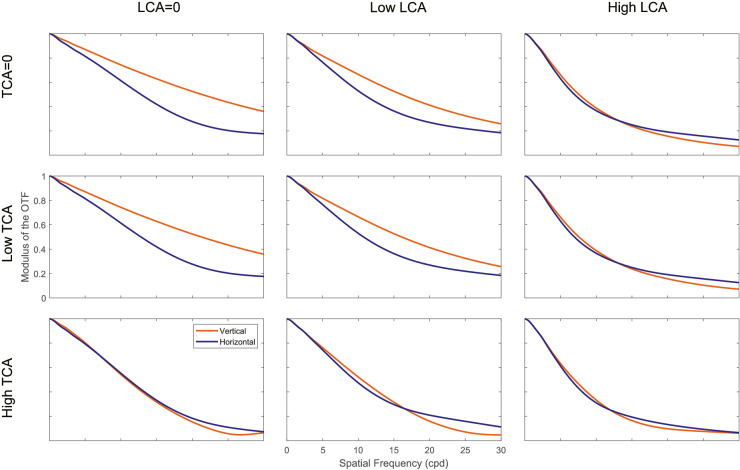
Polychromatic MTF for the white stimulus for one typical subject with increasing amounts of LCA and TCA. Low LCA = −0.998, +0.280 D, and high LCA = −2.554, +1.036 D at λ = 400, 700 nm. Low TCA = 0.4, 0.2 arcmin, and high TCA = 6.3, 3.1 arcmin in the sagittal and transverse meridians from λ = 400–700 nm.

**Figure A1.6.**
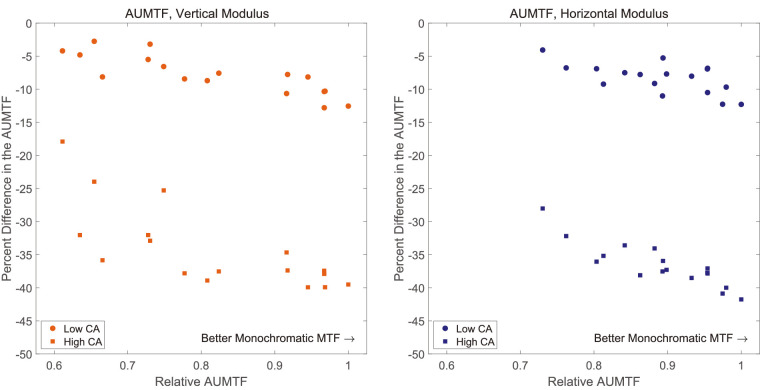
Percent change in the area of the polychromatic MTF as a function of the relative area of the monochromatic MTF for the white stimulus for all 18 eyes.

## Appendix 1. Appendix 1: Modeling chromatic aberration

Here we use optical principles to estimate the expected impact of LCA and/or TCA correction for the stimuli in our experiment. We developed models of LCA and TCA and investigated their effects on vision for typical ranges of human LCA and TCA. We did this for the diffraction-limited eye and for real eyes with typical monochromatic aberrations.

*Modeling LCA.* Thibos and colleagues (1992) developed an optical model of ocular LCA that we use here (as opposed to Equation 1) because it allows us to properly model a range of LCA and TCA. For Emsley's (1948) reduced eye, the axial refractive error *ΔR* is given by

(A1.1) $$\Delta R=\frac{n_{ref}-n\left(\lambda \right)}{r\cdot n_{D}}$$

where *$n_{ref}$* is the refractive index at a reference wavelength (λ = 555 nm), *n(λ)* is the index of the medium, *$n_{D}$* is the index at the model's emmetropic wavelength (589 nm) and is assumed to be 1.333, and *r* is the radius of curvature of the refracting surface, which we assume is 5.55 mm.

To model the variation of the refractive index with wavelength, we use Cornu's hyperbolic formula for the refractive index of water (LeGrand & El Hage, 2013):

(A1.2) $$n\left(\lambda \right)=a+\frac{b}{\lambda -c}$$

where λ is in micrometers, and a = 1.31848, b = 0.006662, and c = 0.1292. Solve Equation A1.2 for λ = 555 nm to obtain *$n_{ref}$*:

(A1.3) $$\;\;\;\;\;\;\;\;n_{ref}=1.31848+\frac{0.006662}{0.555-0.1292}=1.33413$$

Substitute Equations A1.2 and A1.3 into Equation A1.1 to obtain LCA as a function of wavelength:

(A1.4) $$\;\;\;\;\;\;\;\;\Delta R=\frac{n_{ref}-a+\frac{b}{\lambda -c}}{r\cdot n_{D}}=\frac{1.33413-a+\frac{b}{\lambda -c}}{0.00555m\cdot 1.333}$$

LCA is similar in all humans (Atchison & Smith, 2005) but varies nontrivially from one individual to another (Wald & Griffin, 1947; Bedford & Wyszecki, 1957; Ware, 1982; Vinas et al., 2015). As a result, it is desirable to model a range of LCA. This is achieved by attributing variation in LCA to differences in dispersion of the ocular media. Then, with three measures of axial refractive error, one for each display primary, we obtain

(A1.5) $$\Delta R_{r,g,b}=\frac{1.33413-a+\frac{b}{\lambda_{r,g,b}-c}}{0.00555m\cdot 1.333}$$

where Δ$R_{r}$, Δ$R_{g}$, and Δ$R_{b}$ represent the axial refractive error of the red, green, and blue display primaries. We set Δ$R_{g}$ to 0 so that the refractive index at the reference wavelength is constant for all models. Figure A1.1 demonstrates a range of LCA models selected to emulate the range of LCA reported in the literature.

*Modeling TCA.* Thibos developed a model of TCA based on Gullstrand's reduced-eye model (Thibos, 1987; Emsley, 1948; Gullstrand, 1925). Thibos modified the reduced-eye model by incorporating wavelength-dependent refractive indices and placing the limiting aperture behind the refracting surface to maintain the appropriate entrance and exit pupils. For the modified reduced-eye model, analysis begins by constructing the chief ray, which joins a reference point on the object to the center P of the entrance pupil. Then the angle of incidence α and the angle between the chief ray and the optical axis of the eye ε are given by

(A1.6) sinα=|NP|sin(ε)/r

where |NP| is the distance from the nodal point to the center of the entrance pupil (assumed to be 3.98 mm), and r is the radius of curvature of the refracting surface (5.55 mm). The angle of refraction β is computed from Snell's law:

(A1.7) sinβ=sin(α)/n

where n is the refractive index with wavelength dependence given by Equation A1.2. The exit angle γ—the angle that the refracted chief ray makes with respect to the optical axis—is

(A1.8) γ=ε-α+β

The significance of the exit chief ray is that it defines the center of the dioptric blur circle formed in the image plane. Solving Equation A1.6 for α and substituting knowns gives

(A1.9) $$\alpha =\arcsin\left(\frac{3.98}{5.55}\sin\varepsilon \right)$$

Then, solving Equation A1.7 for β and substituting Equation A1.9 yields

(A1.10) $$\beta =\arcsin\left(\frac{1}{n}\left(\frac{3.98}{5.55}\sin\varepsilon \right)\right)$$

Finally, substituting Equations A1.9 and A1.10 into Equation A1.8 gives

(A1.11) $$\begin{array}{ccc} \;\;\;\;\;\;\;\;\;\;\gamma (\lambda ,\varepsilon ) & \;= & \varepsilon -\arcsin\left(\frac{3.98}{5.55}\sin\varepsilon \right)\\ & & \;+\;\arcsin\left(\frac{1}{n(\lambda )}\left(\frac{3.98}{5.55}\sin\varepsilon \right)\right)\;\; \end{array}$$

where n(λ) is given by Equation A1.2. With transverse angular displacement for the red and blue display primaries—γ${}_{r}$ and γ${}_{b}$—the total transverse displacement δ is

(A1.12) $$\begin{array}{ccc} \;\;\;\;\;\;\;\;\;\;\delta & \;= & \gamma_{r}-\gamma_{b}=\varepsilon -\arcsin\left(\frac{3.98}{5.55}\sin\varepsilon \right)\\ & & \;+\;\arcsin\left(\frac{1}{n\left(\lambda_{r}\right)}\left(\frac{3.98}{5.55}\sin\varepsilon \right)\right)\\ & & \;-\;\varepsilon +\arcsin\left(\frac{3.98}{5.55}\sin\varepsilon \right)\\ & & \;-\;\arcsin\left(\frac{1}{n\left(\lambda_{b}\right)}\left(\frac{3.98}{5.55}\sin\varepsilon \right)\right)\\ & \;= & \arcsin\left(\frac{3.98}{5.55}\frac{\sin\varepsilon }{n\left(\lambda_{r}\right)}\right)-\arcsin\left(\frac{3.98}{5.55}\frac{\sin\varepsilon }{n\left(\lambda_{b}\right)}\right) \end{array}$$

where λ${}_{r}$ and λ${}_{b}$ are the wavelengths of the red and blue primaries. With δ determined experimentally and λ${}_{r}$ and λ${}_{b}$ known, Equation A1.12 can be solved numerically to obtain ε. Finally, with ε known, the wavelength- dependent TCA model is obtained by substitution into Equation A1.11. Figure A1.2 demonstrates a range of TCA models chosen to emulate the range of TCA observed in the literature.

*Optical impact of LCA and TCA:* *Spectral weighting function*. A spectral weighting function based on our primaries is needed to estimate the visual impact of TCA and LCA. We measured the spectral power of the primaries ($\phi_{r}$, $\phi_{g}$, $\phi_{b}$) with a radiometrically calibrated spectrometer (PR650; Photo Research Inc., Chatsworth, CA, USA) placed in the pupil plane. The spectral power of each stimulus was estimated as the sum of the spectral power of each primary ϕ = $\phi_{r}$ + $\phi_{g}$ + $\phi_{b}$. The power was weighted by photopic luminosity:

(A1.13) $$\phi_{V(\lambda )}=\phi \cdot V(\lambda )$$

and normalized

(A1.14) $${\tilde{\phi }}_{V(\lambda )}=\phi_{V(\lambda )}/\phi_{o}$$

where $\phi_{o}=\max\left(\phi_{V(\lambda )}\right)$. Spectral samples were taken with 10-nm spacing across the visible spectrum. This process was repeated for each of the three stimuli in the visual acuity experiment: white, purple, and green (Figure A1.3). The relative spectral weights ω(λ) were drawn from the normalized photopic-weighted spectral power distribution.

*Eye model simulation.* Simulation of optical performance was performed using 19 different eye models: one diffraction-limited eye and 18 eyes whose wavefronts were reported in a study with 74 subjects (Cheng et al., 2004). The 18 wavefronts were drawn uniformly from the list of 74 subjects after ordering them by their high-order root-mean-square aberration. The age range was 22–40 years. No gender information was provided. Each dataset was an average of the Zernike coefficients extracted from three high-quality wavefront sensor images. The wavefront chosen to compute the point-spread function (PSF) corresponded to the wavefront with a defocus value that yielded the maximum Strehl ratio and was determined by computing the through-focus PSF and corresponding Strehl ratio in 0.1-D steps. Entrance pupil diameter was set to 4.0 mm. The PSF and MTF were computed using a Fourier method (Nankivil, Raymond, Hofmann, & Neal, 2020). Wavefront estimates for each of the selected wavelengths were obtained assuming three levels of LCA ((0, 0), (−0.998, +0.280), (−2.554, +1.036) D at λ = 400, 700 nm) and three levels of TCA ((0, 0), (0.4, 0.2), and (6.3, 3.1) arcmin in the horizontal and vertical meridians from λ = 400–700 nm). Values of LCA and TCA values were chosen that represent the range of observed subjective LCA and TCA (Wald & Griffin, 1947; Bedford & Wyszecki, 1957; Charman & Jennings, 1976; Ware, 1982; Howarth & Bradley, 1986; Thibos et al., 1992; Vinas et al., 2015; Jiang et al., 2019; Suchkov et al., 2019; Simonet & Campbell, 1990b; Rynders et al., 1995). The polychromatic PSF was then obtained by integrating the ω(λ)-weighted monochromatic point-spread function.

### A.1 Simulation results

*Optical impact of LCA and TCA.* PSF and radial MTF plots are shown for the white stimulus for one typical eye (Figures A1.4 and A1.5). Simulations show that plausible values of LCA and TCA together, or in isolation, cause a substantive decrease in photopic efficacy–weighted retinal-image quality. Typical LCA values have a more detrimental impact on image quality than typical TCA. This concurs with the answer YES to question 7 in Table 1. Comparison of the three stimuli illustrates three intuitive points: (1) Broadband stimuli are more negatively impacted by chromatic aberrations than narrowband stimuli, (2) stimuli with more blue light are more negatively impacted by chromatic aberrations, and (3) even single-primary green stimuli are degraded, although to a lesser extent, by chromatic aberrations.

Perceptual image quality is thought to be largely driven by spatial frequencies of 3–12 cpd (Nankivil, Chen, & Wooley, 2018). Given this, we computed the relative area of modulation amplitudes from 3–12 cpd in the diffraction-limited eye for each of our stimuli with low and high amounts of chromatic aberration. The results are provided are in Table A1.1. Chromatic aberration reduces the area of the photopic efficacy–weighted MTF by ∼7–44% for white, 15–63% for purple, and 3–22% for green stimuli. The percent reductions in the MTF area are calculated by 100(1–x), where x is a value in Table A1.1. With zero TCA, the area of the photopic efficacy–weighted MTF is reduced by approximately 7% to 41%, 15% to 61%, and 3% to 20% due to low and high amounts of LCA, for white, purple, and green stimuli, respectively. With zero LCA, the area of the photopic efficacy–weighted MTF is reduced by ∼0–19%, 0–38%, and 0–4% due to low and high amounts of TCA for white, purple, and green stimuli, respectively. Thus, high amounts of LCA degrade the MTF considerably more than high amounts of TCA, and the addition of high amounts of TCA to a subject with high amounts of LCA does very little to further degrade the efficacy-weighted MTF. This concurs with the answer NO to Questions 8–10 in Table 1.

**Table A1.1.** Relative area under the MTF from 3–12 cpd along the vertical and horizontal meridians for white, purple, and green stimuli for a diffraction-limited eye with a 4-mm entrance pupil.

	Relative area under the MTF (3–12 cpd)					
	Vertical	Horizontal	Vertical	Horizontal	Vertical	Horizontal
	zero LCA	zero LCA	low LCA	low LCA	high LCA	high LCA
	1.0000	1.0000	0.9291	0.9291	0.5869	0.5869
White{ Zero TCA Low TCA High TCA	0.9968	0.9999	0.9281	0.9291	0.5869	0.5869
	0.8076	0.9480	0.7807	0.8912	0.5610	0.5797
	1.0000	1.0000	0.8477	0.8477	0.3870	0.3870
Purple{ Zero TCA Low TCA High TCA	0.9975	0.9995	0.8456	0.8478	0.3870	0.3870
	0.6202	0.8867	0.6143	0.7878	0.3665	0.3815
	1.0000	1.0000	0.9721	0.9721	0.8018	0.8018
Green{ Zero TCA Low TCA High TCA	0.9999	1.0000	0.9719	0.9721	0.8018	0.8018
	0.9567	0.9892	0.9306	0.9625	0.7809	0.7967

**Table A1.2.** Relative area under the MTF from 3–12 cpd along the vertical and horizontal meridians averaged across all eyes.

	Relative area under the MTF (3–12 cpd)					
	Vertical	Horizontal	Vertical	Horizontal	Vertical	Horizontal
	zero LCA	zero LCA	low LCA	low LCA	high LCA	high LCA
	1.0000	1.0000	0.9222	0.9179	0.7166	0.6603
White{ Zero TCA Low TCA High TCA	0.9997	1.0000	0.9215	0.9179	0.7164	0.6603
	0.8443	0.9414	0.7884	0.8691	0.6628	0.6354

The relative area of the modulation amplitudes from 3–12 cpd in typical eyes are shown in Table A1.2 for the white stimuli with low and high amounts of chromatic aberration. The relative impact of chromatic aberration was determined independently for each eye and then averaged across all eyes to obtain the results shown. The results obtained from measured wavefronts were similar to those obtained with the diffraction-limited eye; however, the addition of monochromatic aberrations decreases the relative impact of LCA and TCA.

In order to compare the role of monochromatic aberrations on the impact of chromatic aberrations, the relative area under (AU) the monochromatic MTF was calculated, where unity represents the eye in the dataset with the greatest AUMTF. For each eye, the percent change in the polychromatic AUMTF was calculated, again for the white stimulus only. Comparing these two outputs in a scatterplot illustrates that the decrease in the relative impact of LCA and TCA is more pronounced in subjects with greater amounts of monochromatic aberrations (Figure A1.6). Other researchers have similarly shown that the impact of chromatic aberrations is reduced in the presence of monochromatic aberrations (McLellan, Marcos, Prieto, & Burns, 2002; Ravikumar, Thibos, & Bradley, 2008). Despite this relationship, even in the subject with the most degraded monochromatic MTF, chromatic aberration reduces the area of the MTF from 3–12 cpd substantially: by ∼8–36% for white stimuli.

*Discussion of simulation results.* Estimates of the visual impact of LCA and TCA indicate that (1) typical amounts of foveal TCA do not degrade vision appreciably, but high amounts of TCA do degrade vision; (2) even low amounts of foveal LCA significantly degrade vision; (3) adding TCA to an eye with high amounts of LCA has minimal impact on vision; (4) LCA and/or TCA correction have greater impact for the purple stimulus than for the white stimulus; (5) LCA correction can have a nontrivial impact on vision with green stimuli for subjects with large amounts of LCA; (6) the addition of monochromatic aberrations decreases the relative impact of LCA and TCA; and (7) the decrease in the relative impact of LCA and TCA is more pronounced in subjects with greater amounts of monochromatic aberrations.

## Appendix 2. Appendix 2: Data tables

**Table A2.1.** Longitudinal chromatic aberration data. Units are all in diopters and plus-minus values are standard deviations. Individual data are offset to have a mean of 0 at 533 nm.

	Wavelength					
	468		533		616	
Subject	Left eye	Right eye	Left eye	Right eye	Left eye	Right eye
S1	−0.81 ± 0.08	−0.71 ± 0.22	0 ± 0.08	0 ± 0.27	0.65 ± 0.20	0.65 ± 0.13
S2	−0.41 ± 0.18	−0.43 ± 0.12	0 ± 0.12	0 ± 0.05	0.56 ± 0.05	0.66 ± 0.08
S3	−0.73 ± 0.25	−0.73 ± 0.29	0 ± 0.36	0 ± 0.13	0.51 ± 0.19	0.43 ± 0.20
S4	−0.93 ± 0.13	−0.9 ± 0.41	0 ± 0.23	0 ± 0.35	0.6 ± 0.19	0.45 ± 0.32
S5	−0.58 ± 0.08	−0.48 ± 0.14	0 ± 0.14	0 ± 0.11	0.7 ± 0.09	0.61 ± 0.10
S6	−0.68 ± 0.23	−1.08 ± 0.09	0 ± 0.11	0 ± 0.28	0.76 ± 0.49	0.36 ± 0.30
S7	−0.3 ± 0.38	−0.73 ± 0.09	0 ± 0.23	0 ± 0.35	0.9 ± 0.13	0.51 ± 0.16

**Table A2.2.** Transverse chromatic aberration data. Positive TCA values indicate that a blue or green central target appears displaced to the right and upward relative to the red. Plus-minus values are standard deviations. The number of trials for the TCA estimates is 6 for all eyes and all conditions except for S1 right (36), S2 left (42), and S3 right (36).

			Left-eye TCA		Right-eye TCA	
Subject	ACL	Color	Hor	Vert	Hor	Vert
S1	0	B	0.02 ± 0.09	−1.51 ± 0.17	0.71 ± 0.23	0.51 ± 0.21
		G	−0.19 ± 0.19	−1.26 ± 0.19	0.50 ± 0.17	0.35 ± 0.11
	1	B	−1.04 ± 0.19	−0.71 ± 0.18	2.25 ± 0.25	1.04 ± 0.24
		G	−0.53 ± 0.13	−0.44 ± 0.12	1.29 ± 0.16	0.62 ± 0.12
S2	0	B	0.28 ± 0.46	−0.07 ± 0.56	−0.70 ± 0.12	0.81 ± 0.17
		G	0.41 ± 0.28	−0.22 ± 0.48	−0.53 ± 0.09	0.31 ± 0.11
	1	B	−0.21 ± 0.47	0.36 ± 0.25	0.93 ± 0.17	0.52 ± 0.17
		G	−0.13 ± 0.29	0.23 ± 0.19	0.58 ± 0.18	0.46 ± 0.21
S3	0	B	0.40 ± 0.41	1.67 ± 0.24	1.39 ± 0.36	−0.45 ± 0.61
		G	0.02 ± 0.26	1.15 ± 0.11	0.79 ± 0.32	−0.07 ± 0.65
	1	B	−0.87 ± 0.37	0.01 ± 0.14	1.88 ± 0.23	0.31 ± 0.67
		G	−0.47 ± 0.42	0.22 ± 0.56	1.20 ± 0.18	0.16 ± 0.47
S4	0	B	−0.30 ± 0.50	0.04 ± 0.53	−0.53 ± 0.28	−0.77 ± 0.38
		G	−0.23 ± 0.75	−0.17 ± 0.19	−0.25 ± 0.23	−0.65 ± 0.64
	1	B	−2.02 ± 0.81	2.40 ± 0.74	3.33 ± 0.48	0.37 ± 0.86
		G	−0.68 ± 0.60	1.21 ± 0.60	1.81 ± 0.24	0 ± 0.52
S5	0	B	−1.60 ± 0.44	−1.37 ± 0.27	−1.15 ± 0.53	−0.65 ± 0.83
		G	−0.61 ± 0.54	−0.46 ± 0.34	−0.41 ± 0.33	0.11 ± 0.23
	1	B	−2.78 ± 0.18	1.55 ± 0.50	1.60 ± 0.31	1.94 ± 0.42
		G	0.94 ± 0.28	0.85 ± 0.23	0.76 ± 0.24	1.44 ± 0.18
S6	0	B	−1.93 ± 0.65	0.98 ± 0.29	−1.18 ± 0.56	−0.18 ± 0.28
		G	−1.05 ± 0.41	0.64 ± 0.30	−0.85 ± 0.21	0.32 ± 0.31
	1	B	−2.22 ± 0.34	−0.43 ± 0.33	4.24 ± 0.74	0 ± 0.55
		G	−0.85 ± 0.73	−0.51 ± 0.26	1.88 ± 0.40	−0.01 ± 0.65
S7	0	B	−0.93 ± 0.42	−1.18 ± 0.19	−0.65 ± 0.41	0.49 ± 0.45
		G	0.11 ± 0.46	−0.17 ± 0.43	−0.58 ± 0.42	0.30 ± 0.38
	1	B	−1.04 ± 0.23	0.16 ± 0.39	4.29 ± 0.22	−0.24 ± 0.44
		G	−0.43 ± 0.27	0.20 ± 0.15	2.19 ± 0.21	0.06 ± 0.27

**Table A2.3.** LogMAR visual acuities for all 10 conditions. **A** refers to the LCA correction (0 = LCA present, 1 = LCA corrected). **T** refers to the TCA correction (0 = TCA present, 1 = TCA corrected). The three rows for each subject contain the upper 90% CI, the mean, and the lower 90% CI.

	Green	Purple	Purple	White	White	Green	Purple	Purple	White	White
Subject	A0	A0T0	A0T1	A0T0	A0T1	A1	A1T0	A1T1	A1T0	A1T1
S1 upper 90% CI	−0.02	0.06	0.05	0.05	0.03	0.01	0.11	0.07	0.06	0.01
S1	−0.06	0.03	0.01	0.01	−0.01	−0.05	0.06	0	0.01	−0.02
S1 lower 90% CI	−0.10	0.01	−0.01	0.02	−0.04	−0.09	0.02	−0.06	−0.04	−0.04
S2 upper 90% CI	0.19	0.28	0.29	0.24	0.21	0.10	0.18	0.17	0.15	0.15
S2	0.14	0.20	0.20	0.16	0.15	0.04	0.13	0.11	0.10	0.09
S2 lower 90% CI	0.11	0.15	0.14	0.11	0.11	−0.01	0.09	0.05	0.07	0.03
S3 upper 90% CI	0.07	0.15	0.14	0.17	0.13	0.06	0.15	0.07	0.18	0.08
S3	0.02	0.09	0.08	0.11	0.09	0	0.07	0.01	0.08	0.02
S3 lower 90% CI	−0.03	0.05	0.02	0.07	0.05	−0.04	0	−0.03	0.02	−0.04
ALL upper 90% CI	0.11	0.20	0.18	0.18	0.14	0.03	0.13	0.09	0.13	0.07
ALL	0.05	0.13	0.11	0.11	0.09	0	0.09	0.05	0.07	0.03
ALL lower 90% CI	0	0.08	0.07	0.05	0.05	−0.03	0.05	0.01	0.02	0

**Table A2.4.** Table of red vs. blue horizontal TCA values and binocular disparity values. Positive TCA values indicate that the central blue target appears displaced to the right relative to the red. Computed disparity is the difference between the left- and right-eye TCA offsets. Measured disparity is disparity determined in the experiment to equate the perceived depths of the red and blue bars. For the TCA, the plus-minus values are standard deviations. For the disparities, the 90% confidence range is provided in the square brackets. The number of trials for the TCA estimates is 6 for all eyes and all conditions.

		Horizontal TCA		Binocular disparity	
Subject	ACL	Left	Right	Computed	Measured
S1	0	−0.74 ± 0.09	0.59 ± 0.19	1.34 ± 0.22	0.11 [−0.34 + 0.31]
	1	−1.82 ± 0.19	1.65 ± 0.15	3.47 ± 0.25	2.43 [−0.45 +0.44]
S2	0	−0.48 ± 0.16	−1.03 ± 0.12	−0.55 ± 0.21	−1.27 [−0.25 +0.23]
	1	−1.30 ± 0.21	0.26 ± 0.17	1.57 ± 0.27	1.14 [−0.15 +0.16]
S3	0	−0.36 ± 0.41	1.22 ± 0.05	1.59 ± 0.41	0.84 [−0.49 +0.50]
	1	−1.67 ± 0.37	1.15 ± 0.14	2.82 ± 0.40	2.86 [−0.21 +0.23]
S4	0	0.01 ± 0.50	0.19 ± 0.28	0.17 ± 0.58	1.78 [−0.19 +0.20]
	1	−3.03 ± 0.81	0.97 ± 0.48	4.01 ± 0.95	1.61 [−0.21 +0.17]
S5	0	−1.28 ± 0.44	−0.42 ± 0.53	0.86 ± 0.69	1.35 [−0.43 +0.51]
	1	−3.74 ± 0.18	−0.44 ± 0.31	3.30 ± 0.36	3.93 [−0.20 +0.19]
S6	0	−1.61 ± 0.65	−0.46 ± 0.56	1.15 ± 0.87	0.63 [−0.33 +0.31]
	1	−3.22 ± 0.34	1.93 ± 0.74	5.16 ± 0.81	5.36 [−0.42 +0.30]
S7	0	−0.61 ± 0.42	0.07 ± 0.41	0.69 ± 0.59	1.92 [−0.48 +0.53]
	1	−1.90 ± 0.23	1.99 ± 0.22	3.89 ± 0.32	3.96 [−0.24 +0.39]
